# Efficacy of ChAdOx1 nCoV-19 (AZD1222) vaccine against SARS-CoV-2 variant of concern 202012/01 (B.1.1.7): an exploratory analysis of a randomised controlled trial

**DOI:** 10.1016/S0140-6736(21)00628-0

**Published:** 2021-04-10

**Authors:** Katherine R W Emary, Tanya Golubchik, Parvinder K Aley, Cristina V Ariani, Brian Angus, Sagida Bibi, Beth Blane, David Bonsall, Paola Cicconi, Sue Charlton, Elizabeth A Clutterbuck, Andrea M Collins, Tony Cox, Thomas C Darton, Christina Dold, Alexander D Douglas, Christopher J A Duncan, Katie J Ewer, Amy L Flaxman, Saul N Faust, Daniela M Ferreira, Shuo Feng, Adam Finn, Pedro M Folegatti, Michelle Fuskova, Eva Galiza, Anna L Goodman, Catherine M Green, Christopher A Green, Melanie Greenland, Bassam Hallis, Paul T Heath, Jodie Hay, Helen C Hill, Daniel Jenkin, Simon Kerridge, Rajeka Lazarus, Vincenzo Libri, Patrick J Lillie, Catherine Ludden, Natalie G Marchevsky, Angela M Minassian, Alastair C McGregor, Yama F Mujadidi, Daniel J Phillips, Emma Plested, Katrina M Pollock, Hannah Robinson, Andrew Smith, Rinn Song, Matthew D Snape, Rebecca K Sutherland, Emma C Thomson, Mark Toshner, David P J Turner, Johan Vekemans, Tonya L Villafana, Christopher J Williams, Adrian V S Hill, Teresa Lambe, Sarah C Gilbert, Merryn Voysey, Maheshi N Ramasamy, Andrew J Pollard, COVID-19 Genomics UK consortium, the AMPHEUS Project, Oxford COVID-19 Vaccine Trial Group

**Affiliations:** a Oxford Vaccine Group, Department of Paediatrics, University of Oxford, Oxford, UK; b Jenner Institute, Nuffield Department of Medicine, University of Oxford, Oxford, UK; c Clinical BioManufacturing Facility, University of Oxford, Oxford, UK; d Wellcome Centre for Human Genetics, Nuffield Department of Medicine, University of Oxford, Oxford, UK; e Big Data Institute, Nuffield Department of Medicine, University of Oxford, Oxford, UK; f Wellcome Sanger Institute, Wellcome Genome Campus, Hinxton, UK; g COVID-19 Genomics UK, Department of Medicine, University of Cambridge, Cambridge, UK; h National Infection Service, Public Health England, Salisbury, UK; i Department of Clinical Sciences, Liverpool School of Tropical Medicine and Liverpool University Hospitals NHS Foundation Trust, Liverpool, UK; j UK Biocentre, Milton Keynes, UK; k Department of Infection, Immunity and Cardiovascular Disease, University of Sheffield, Sheffield, UK; l Department of Infection and Tropical Medicine, Sheffield Teaching Hospitals NHS Foundation Trust, Sheffield, UK; m Department of Infection and Tropical Medicine, Newcastle upon Tyne Hospitals NHS Foundation Trust, Newcastle upon Tyne, UK; n Translational and Clinical Research Institute, Immunity and Inflammation Theme, Newcastle University, Newcastle upon Tyne, UK; o NIHR Southampton Clinical Research Facility and Biomedical Research Centre, University Hospital Southampton NHS Foundation Trust; p Faculty of Medicine and Institute for Life Sciences, University of Southampton, Southampton, UK; q University Hospitals Bristol and Weston NHS Foundation Trust, Bristol, UK; r St George's Vaccine Institute, St George's, University of London, London, UK; s Department of Infection, Guy's and St Thomas' NHS Foundation Trust, St Thomas' Hospital, London, UK; t MRC Clinical Trials Unit, University College London, London, UK; u NIHR/Wellcome Trust Clinical Research Facility, University Hospitals Birmingham NHS Foundation Trust, Birmingham, UK; v University of Glasgow, Glasgow, UK; w Lighthouse Laboratory in Glasgow, Queen Elizabeth University Hospital, Glasgow, UK; ah Severn Pathology, North Bristol NHS Trust, Bristol, UK; y NIHR UCLH Clinical Research Facility, London, UK; z NIHR UCLH Biomedical Research Centre, London, UK; aa Hull University Teaching Hospitals NHS Trust, Hull, UK; ab London Northwest University Healthcare, Harrow, UK; ac NIHR Imperial Clinical Research Facility, London, UK; ad NIHR Imperial Biomedical Research Centre, London, UK; ae College of Medical, Veterinary & Life Sciences, Glasgow Dental Hospital and School, University of Glasgow, Glasgow, UK; af Clinical Infection Research Group, Regional Infectious Diseases Unit, Western General Hospital, Edinburgh, UK; ag MRC University of Glasgow Centre for Virus Research, Glasgow, UK; ai Heart Lung Research Institute, Department of Medicine, University of Cambridge, Cambridge, UK; aj NIHR Cambridge Clinical Research Facility, Cambridge, UK; ak Cambridge University Hospital and Royal Papworth NHS Foundation Trusts, Cambridge, UK; al University of Nottingham, Nottingham, UK; am Nottingham University Hospitals NHS Trust, Nottingham, UK; an AstraZeneca BioPharmaceuticals, Gaithersburg, MD, USA; ao Public Health Wales, Cardiff, UK; ap Aneurin Bevan University Health Board, Newport, UK; aq Department of Infectious Diseases, Queen Elizabeth University Hospital, Glasgow, UK

## Abstract

**Background:**

A new variant of SARS-CoV-2, B.1.1.7, emerged as the dominant cause of COVID-19 disease in the UK from November, 2020. We report a post-hoc analysis of the efficacy of the adenoviral vector vaccine, ChAdOx1 nCoV-19 (AZD1222), against this variant.

**Methods:**

Volunteers (aged ≥18 years) who were enrolled in phase 2/3 vaccine efficacy studies in the UK, and who were randomly assigned (1:1) to receive ChAdOx1 nCoV-19 or a meningococcal conjugate control (MenACWY) vaccine, provided upper airway swabs on a weekly basis and also if they developed symptoms of COVID-19 disease (a cough, a fever of 37·8°C or higher, shortness of breath, anosmia, or ageusia). Swabs were tested by nucleic acid amplification test (NAAT) for SARS-CoV-2 and positive samples were sequenced through the COVID-19 Genomics UK consortium. Neutralising antibody responses were measured using a live-virus microneutralisation assay against the B.1.1.7 lineage and a canonical non-B.1.1.7 lineage (Victoria). The efficacy analysis included symptomatic COVID-19 in seronegative participants with a NAAT positive swab more than 14 days after a second dose of vaccine. Participants were analysed according to vaccine received. Vaccine efficacy was calculated as 1 − relative risk (ChAdOx1 nCoV-19 *vs* MenACWY groups) derived from a robust Poisson regression model. This study is continuing and is registered with ClinicalTrials.gov, NCT04400838, and ISRCTN, 15281137.

**Findings:**

Participants in efficacy cohorts were recruited between May 31 and Nov 13, 2020, and received booster doses between Aug 3 and Dec 30, 2020. Of 8534 participants in the primary efficacy cohort, 6636 (78%) were aged 18–55 years and 5065 (59%) were female. Between Oct 1, 2020, and Jan 14, 2021, 520 participants developed SARS-CoV-2 infection. 1466 NAAT positive nose and throat swabs were collected from these participants during the trial. Of these, 401 swabs from 311 participants were successfully sequenced. Laboratory virus neutralisation activity by vaccine-induced antibodies was lower against the B.1.1.7 variant than against the Victoria lineage (geometric mean ratio 8·9, 95% CI 7·2–11·0). Clinical vaccine efficacy against symptomatic NAAT positive infection was 70·4% (95% CI 43·6–84·5) for B.1.1.7 and 81·5% (67·9–89·4) for non-B.1.1.7 lineages.

**Interpretation:**

ChAdOx1 nCoV-19 showed reduced neutralisation activity against the B.1.1.7 variant compared with a non-B.1.1.7 variant in vitro, but the vaccine showed efficacy against the B.1.1.7 variant of SARS-CoV-2.

**Funding:**

UK Research and Innovation, National Institute for Health Research (NIHR), Coalition for Epidemic Preparedness Innovations, NIHR Oxford Biomedical Research Centre, Thames Valley and South Midlands NIHR Clinical Research Network, and AstraZeneca.

## Introduction

The COVID-19 pandemic continues to cause considerable mortality, placing a substantial burden on health-care systems around the world and having profound social and economic consequences due to the measures implemented to control the SARS-CoV-2 virus. A number of SARS-CoV-2 vaccines have shown efficacy in large-scale phase 3 trials.^1^, ^2^, ^3^, ^4^, ^5^, ^6^ Although the vaccine platforms differ, most use the surface spike glycoprotein of SARS-CoV-2 as the key antigenic target for the generation of binding and neutralising antibodies and T cells, and use an antigen coding sequence based on the originally identified Wuhan lineage virus (GenBank accession number M908947). Several vaccines have now been licensed for emergency use by individual countries and large-scale vaccination programmes are underway with the anticipation that vaccination will be a key component of future disease control.

**Research in context**

**Evidence before this study**

We searched PubMed for research articles published from database inception until Feb 1, 2021, with no language restrictions, using the terms “SARS-CoV-2” AND “B.1.1.7” OR “VUI-202012/01” OR “VOC-202012/01” OR “Kent”. At the time of the search, no peer-reviewed publications were available on the efficacy of sera from vaccinees to neutralise B.1.1.7 lineage viruses. Preprint articles using convalescent sera suggest that neutralisation activity against pseudovirus expressing B.1.1.7 spike protein could be reduced compared with activity against pseudovirus expressing wild-type spike protein. Preliminary data using sera of vaccinees who were immunised with mRNA vaccines (Pfizer–BioNTech or Moderna) and protein vaccines (Novavax) found either no or a modest reduction in neutralisation activity against pseudoviruses with either spike protein with individual mutations found in B.1.1.7 or whole B.1.1.7 spike protein containing all lineage-defining mutations. Several vaccine developers have published peer-reviewed interim efficacy results against symptomatic COVID-19 disease, and others have reported preliminary efficacy results in press releases. At the time of searching, no peer-reviewed publications were available on the efficacy of a SARS-CoV-2 vaccine against the lineage B.1.1.7. However, a Novavax press release suggests an adjuvanted protein vaccine has vaccine efficacy of 85·6% against the UK B.1.1.7 lineage in a post-hoc analysis.

**Added value of this study**

These are the first published data on the clinical efficacy of a vaccine against the novel B.1.1.7 variant and non-B.1.1.7 lineages of SARS-CoV-2. ChAdOx1 nCoV-19 was efficacious against both the B.1.1.7 variant and non-B.1.1.7 variants. Furthermore, vaccination reduced viral load and length of NAAT positivity against both B.1.1.7 and non-B.1.1.7 lineages.

**Implications of all the available evidence**

The ChAdOx1 nCoV-19 vaccine has been given emergency use authorisation in multiple countries, including the UK. The B.1.1.7 variant is currently responsible for the majority of COVID-19 disease in the UK. These findings support the ongoing use of ChAdOx1 nCoV-19 in mass vaccination programmes to both prevent symptomatic B.1.1.7 disease and reduce the opportunity for viral transmission.

While vaccine trials were underway in 2020, novel lineages of SARS-CoV-2 were identified globally.^7^, ^8^, ^9^ Especially in populations with high levels of natural or vaccine-induced immunity, variants that can evade human immune responses are likely to arise. A novel SARS-CoV-2 variant, designated variant of concern 202012/01 (also known as lineage B.1.1.7),^9^ was identified in Kent, UK, in late 2020, and accounted for an expanding proportion of cases at that time, particularly in the southeast and east of England.^9^ Whereas most SARS-CoV-2 lineages show few mutations, B.1.1.7 has accrued 23 mutations across the genome, including a non-synonymous mutation affecting the spike protein, N501Y (Asn501Tyr), which might increase ACE2 receptor binding affinity,^10^ the spike deletion 69–70del associated with viral escape in immunocompromised individuals,^9^ and the non-synonymous mutation P681H (Pro681His) affecting the furin cleavage site between the S1 and S2 subunits of spike protein, associated with in-vitro enhanced membrane fusion of infected cells.^9^, ^11^ N501Y is also seen in the B.1.351 and P.1 lineages, which are also variants of concern.^7^, ^8^

The national community testing system in the UK (known as Pillar 2) is delivered by a small number of laboratories, many of which are using a ThermoFisher TaqPath three-gene nucleic acid amplification test (NAAT). 69–70del causes a loss of two amino acids at positions 69 and 70 and thereby causes a reproducible S gene dropout in this assay, referred to as S gene target failure (SGTF).^12^ Although other variants circulating in the UK contain this mutation, since Nov 30, 2020, 95% of all UK Pillar 2 69–70del sequences were due to the B.1.1.7 lineage,^13^ permitting the use of the SGTF result as a proxy marker for this variant between Nov 30, 2020, and Jan 15, 2021.^14^, ^15^ Associations between SGTF (or mutations present in B.1.1.7) and higher viral loads^16^, ^17^ and increased risk of transmission^14^ have already been shown. The B.1.1.7 lineage could result in an additive increase in the basic reproductive number (R_0_) of between 0·4 and 0·7 compared with non-B.1.1.7 lineages^14^, ^18^ in the current UK context, although the mechanisms for increased transmission are complex. Importantly, UK public health measures in place to control the virus failed to control spread of SGTF-containing lineages but were effective for those lineages without SGTF.^14^ Several unpublished, independent analyses suggest that infections associated with SGTF on PCR from upper respiratory samples might be associated with an increased fatality rate compared with non-SFTF-associated infections.^13^, ^19^

The emergence of a variant with multiple mutations also raises concerns regarding the efficacy of natural infection-derived immunity to prevent reinfection, as well as regarding vaccine efficacy. Assessing immune evasion by B.1.1.7 in vivo is challenging given the timing of the emergence of this variant (approximately 8 months after the first wave of infections in the UK) combined with the finding in health-care workers that after primary infection, the observed median protective effect was at least 5 months.^20^ However, reinfection with B.1.1.7 and subsequent critical illness has been reported.^21^

Amino acid 501 of the SARS-CoV-2 spike protein sits within the receptor binding domain (RBD) of the S1 subunit. The RBD binds to ACE2 receptors and permits viral entry into host cells and is a key target for neutralising antibodies. Neutralising antibodies outcompete binding to the RBD, thereby preventing infection from both pseudovirus and wild-type virus.^22^ Early in-silico analysis suggests that the mutations present in B.1.1.7 might confer only small changes in epitope signal.^23^ Early studies assessing the neutralising ability of sera from convalescent patients and vaccinees against pseudoviruses expressing some mutations present in B.1.1.7 or the entire B.1.1.7 spike protein suggest no or little reduction in neutralisation compared with older circulating viral variants.^24^, ^25^, ^26^, ^27^, ^28^ Early reports suggest that vaccine efficacy might not be reduced against this variant.^4^

The B.1.1.7 variant in the UK arose while there was a low level of population immunity from natural infection and before vaccine programmes had begun, so the variant is likely to have been selected for improved ACE2 receptor binding and transmissibility, rather than as a result of vaccine-induced immunity. Furthermore, multiple epitopes are recognised by neutralising antibody and other antibody functions^29^ and binding might occur at non-RBD epitopes in the spike protein, which could provide protection even with the presence of the mutations recognised in B.1.1.7. T-cell responses could also provide protection against infection and have been shown after vaccination with the mRNA^30^, ^31^ and viral vector vaccines^32^ that are currently available in the UK.

Previously, we reported the efficacy of the simian adenoviral vectored vaccine ChAdOx1 nCoV-19 (AZD1222) in randomised controlled trials in Brazil and the UK,^3^ in analyses done before the spread of the B.1.1.7 variant across the UK. In this study, we provide both an in-vitro analysis of vaccine-induced neutralising antibody responses against B.1.1.7 and an analysis of the clinical efficacy of ChAdOx1 nCoV-19 against disease caused by the B.1.1.7 variant of concern, using data from the UK. As novel variants are identified, new targeted vaccines might be needed in future public health programmes as booster doses. We also present data on the immune response to ChAdOx1 nCoV-19 in people who previously received other ChAdOx1-vectored vaccines.

## Methods

### Study design and participants

COV002 is a single-blind, multicentre, randomised phase 2/3 trial assessing the safety and efficacy of the ChAdOx1 nCoV-19 vaccine. Safety, immunogenicity, and efficacy data, including the full protocol and statistical analysis plan have been previously published in detail.^3^, ^29^, ^33^, ^34^

For the current analysis, only participants in efficacy cohorts (n=8534) were included. Briefly, these were adults aged 18 years and older, enrolled at 19 study sites in England, Wales, and Scotland. Enrolment targeted participants in occupations with potentially high SARS-CoV-2 exposure, such as those in health and social care settings. Participants were randomly assigned in a 1:1 ratio to receive standard-dose ChAdOx1 nCoV-19 vaccine (5 × 10^10^ viral particles) or a meningococcal group A, C, W, and Y conjugate vaccine (MenACWY) as control. A subset of participants (n=2773) received a low-dose vaccine (2·2 × 10^10^ viral particles) as their first dose or control, as previously described.^3^

This study was approved in the UK by the Medicines and Healthcare products Regulatory Agency (MHRA), reference 21584/0428/001–0001, and the South-Central Berkshire Research Ethics Committee, reference 20/SC/0179.

### Procedures

Participants were reminded weekly by email or text message to contact the trial team if they developed COVID-19 symptoms, including at least one of cough, a fever of 37·8°C or higher, shortness of breath, anosmia, or ageusia. Participants who met criteria for symptomatic testing underwent clinical assessment and nasopharyngeal and oral swabbing at their local clinical site. Samples were processed using MHRA-derogated NAAT assays within National Health Service (NHS) diagnostic laboratories accredited by the UK Accreditation Service for each study site.

Additionally, participants were asked to provide weekly self-collected nose and throat swabs for NAAT testing from 1 week after first vaccination using home test kits provided by the Department of Health and Social Care. Symptoms in these participants were not routinely assessed because swabs were done at home and sent for testing by mail. Home testing kits were processed at laboratories designated by the Department of Health and Social Care. The Glasgow, Milton Keynes, and Alderley Park Lighthouse Laboratories processed the majority of swab samples from weekly home test kits (98·3% to Jan 14, 2021) and exclusively used the ThermoFisher TaqPath assay (Thermo Fisher Scientific, Waltham, MA, USA; target genes ORF1ab, S, and N) for viral detection. Participants were directly informed of their results by text and email from the NHS. Results from trial-barcoded swabs were provided to the trial team by NHS Digital. Safety data were reviewed regularly by an independent data safety monitoring board.

For immunogenicity assays, sera from vaccinated participants were tested using a live SARS-CoV-2 microneutralisation assay (evaluating ND_50_, the titre at which 50% virus neutralisation is achieved), at Public Health England (Porton Down, UK), as described previously.^33^ The B.1.1.7 lineage and a canonical non-B.1.1.7 Victoria lineage (BetaCoV/Australia/VIC01/2020) were used in neutralising assays.

Humoral responses at baseline and after vaccination in recipients of a different previous ChAdOx1 vaccine were assessed using a standardised total IgG ELISA against trimeric SARS-CoV-2 spike protein, as previously described.^33^

SARS-CoV-2 RNA from trial participant home test kits and diagnostic swabs was identified at Lighthouse Laboratories and 15 of 19 study site laboratories. Samples were batched at the point of testing, and consolidated batches were sent for sequencing and genome assembly to COVID-19 Genomics UK partner laboratories. The majority of sequences (73%) during the study period were generated by Oxford Viromics (Wellcome Centre for Human Genetics, University of Oxford, Oxford, UK) using the quantitative veSEQ approach.^35^, ^36^ Controls were checked to ensure no evidence of amplification in the negative tests and that expected RNA quantification was consistent with cycle threshold (Ct) values provided by the testing laboratories. All samples were processed by laboratory staff who were masked to vaccine allocation.

Viral genomes were assembled as previously described,^35^ and variant frequencies were computed using shiver (tools/AnalysePileup.py),^37^ with the default settings of no base alignment quality and maximum pileup depth of 1 000 000. Lineages were assigned by Pangolin version 2.1.7 (lineages version 2021–02–12) using the determined consensus genome for each sequenced sample. If the proportion of gaps was greater than 50% (the recommended minimum for Pangolin), lineages were assigned on the basis of presence of lineage-defining mutations in the spike gene.^9^ Samples that could not be assigned a lineage by either method were classified as unknown. Consensus sequences were aligned using MAFFT version 7.402.^38^ Phylogenetic reconstruction was performed on the alignment consisting of consensus sequences rooted with the Wuhan-Hu-1 reference sequence (RefSeq NC_045512), using IQ-TREE version 1.6.12,^39^ with the generalised time reversible + FreeRate model and 1000 bootstrap replicates.

### Outcomes

The primary outcome was symptomatic COVID-19 disease, defined as a positive NAAT result on an upper airway swab in a participant with at least one symptom, including cough, fever of 37·8°C or higher, shortness of breath, anosmia, or ageusia.

Asymptomatic infections and those with unknown symptoms detected through weekly swabbing were a secondary outcome. Any NAAT postive infection was a secondary outcome and consisted of primary symptomatic cases, non-primary symptomatic cases (those with other symptoms such as nausea or diarrhoea), asymptomatic cases, and cases for which symptoms were unknown. Additional exploratory outcomes included Ct values from NAATs as an inverse proxy for viral load, and the number of weeks positive on any NAAT.

### Statistical analysis

Cases were included in efficacy analyses from randomised participants enrolled in phase 3 efficacy cohorts that occurred between Oct 1, 2020, and Jan 14, 2021. Participants were analysed according to vaccine received. Both those receiving two standard doses (SD/SD group), or a low dose followed by a standard dose (LD/SD group) are included. Single-dose recipients were excluded. Cases were excluded if they occurred fewer than 15 days after the second dose of vaccine or occurred in participants who were not seronegative on a SARS-CoV-2 N protein assay at baseline.

The ChAdOx1 nCoV-19 vaccine was licensed for use in the UK on Dec 30, 2020, with priority groups for vaccine roll-out (ie, older adults and front-line health-care workers) identified to prevent mortality and support the NHS and social care system. The majority of participants in the trial were recruited from these high-exposure populations and were therefore eligible for vaccination under the government NHS coronavirus vaccine programme. Participants who were unblinded in order to receive a vaccine through the government COVID-19 vaccination scheme were included in the analysis up until the day of their unblinding, and any cases after that were not included in this analysis. All endpoints were reviewed for inclusion by an independent, blinded adjudication committee.

Vaccine efficacy was calculated as 1 – relative risk (ChAdOx1 nCoV-19 *vs* MenACWY groups) calculated from a robust Poisson model in SAS version 9.4. The model contained terms for treatment group and vaccine group (LD/SD or SD/SD). The logarithm of the period at risk was used as an offset variable in the model to adjust for volunteers having different follow-up times during which the events occurred. The code for model fitting is provided in the appendix (p 12).

To determine whether vaccination with ChAdOx1 nCoV-19 was associated with reduced viral load, NAAT Ct values, as a proxy for viral load, were analysed from weekly swabs from home test kits processed in Lighthouse Laboratories. Ct values for swabs done at symptomatic visits were not available. The minimum Ct value across the N and ORF1ab genes from each NAAT was computed for each swab and the minimum Ct value across all positive swabs for each participant was compared between vaccine groups using a Wilcoxon rank sum test. To determine whether vaccination was associated with a reduced number of weeks of NAAT positivity, the number of weeks from the first NAAT positive swab until the last NAAT positive swab during the NAAT-positive period was calculated. Three consecutive NAAT negative swabs were considered to indicate the end of the NAAT-positive period. The number of weeks positive was compared between vaccine groups using a Wilcoxon rank sum test.

Neutralising titres from a live-virus microneutralisation assay were available from sera tested against both the B.1.1.7 lineage and the Victoria lineage. Data were log_2_-transformed to achieve a normal distribution and the geometric mean ratio (Victoria/B.1.1.7) was computed to show the change in neutralising potential, and log_2_ titres were compared using a paired *t* test.

Anti-SARS-CoV-2 spike IgG antibody titres (tested by ELISA) in individuals who had received a previous different ChAdOx1 vaccine were compared with those who received two standard doses of ChAdOx1 nCoV-19 at 14 and 28 days after the first dose, and 28 days after the second dose using Wilcoxon rank sum test.

Baseline characteristics were compared between individuals with B.1.1.7 versus non-B.1.1.7 variants using χ^2^ and Fisher's exact tests for binary variables, a Wilcoxon rank sum test for body-mass index, and Cochran-Armitage tests for ordinal age groups and prime-boost intervals.

This study is registered with ClinicalTrials.gov, NCT04400838, and ISRCTN, 15281137.

### Role of the funding source

AstraZeneca reviewed the data from the study and the final manuscript before submission, but the academic authors retained editorial control. All other funders of the study had no role in study design, data collection, data analysis, data interpretation, or writing of the report.

## Results

Participants in efficacy cohorts were recruited between May 31 and Nov 13, 2020, and received booster doses between Aug 3 and Dec 30, 2020. Of 8534 participants in the primary efficacy cohort, 6636 (78%) were aged 18–55 years, 5065 (59%) were female, and 7863 (92%) were White. 5623 (66%) participants worked in a health or social care setting (appendix p 2).

**Figure 1:**
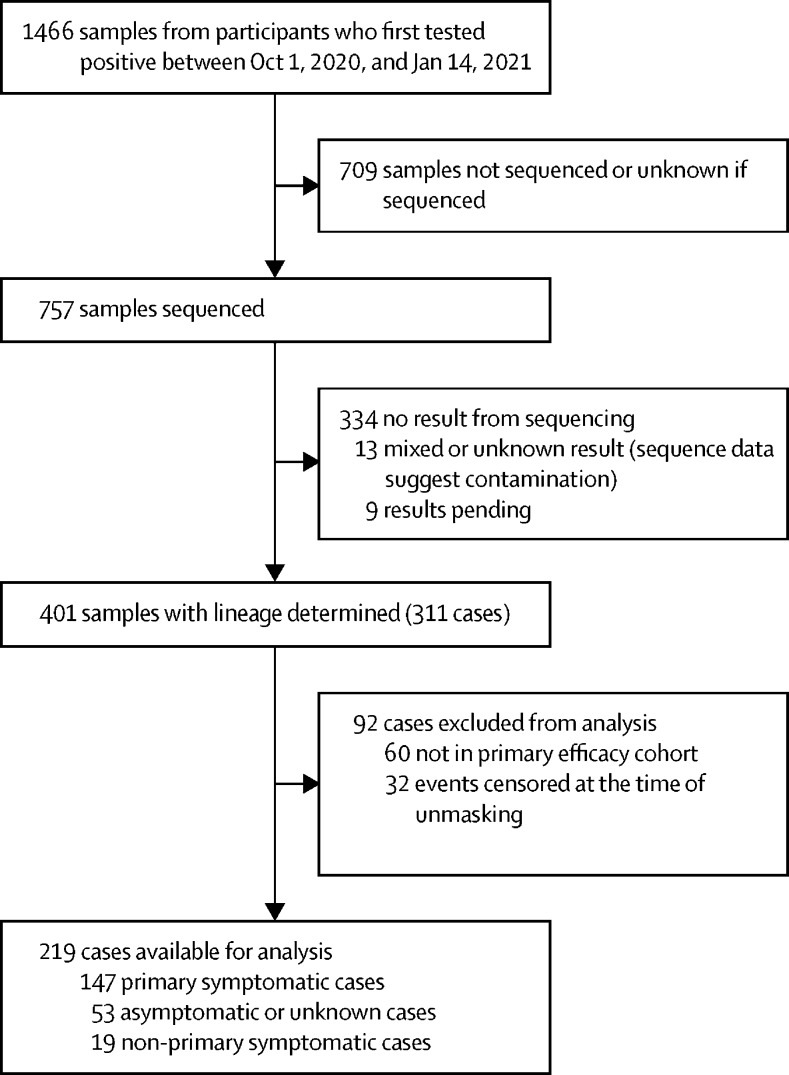
Flow diagram of swabs included in the analysis

Between Oct 1, 2020, and Jan 14, 2021, 520 participants developed SARS-CoV-2 infection. 1466 NAAT positive nose and throat swabs were collected from these participants during the trial. Sequences were available from 401 of these swabs, representing 311 cases. 219 of these cases occurred in participants in the primary efficacy cohort and before unblinding, of which 147 were cases of primary symptomatic COVID-19 and 53 were asymptomatic or had unknown symptoms (figure 1).

**Figure 2:**
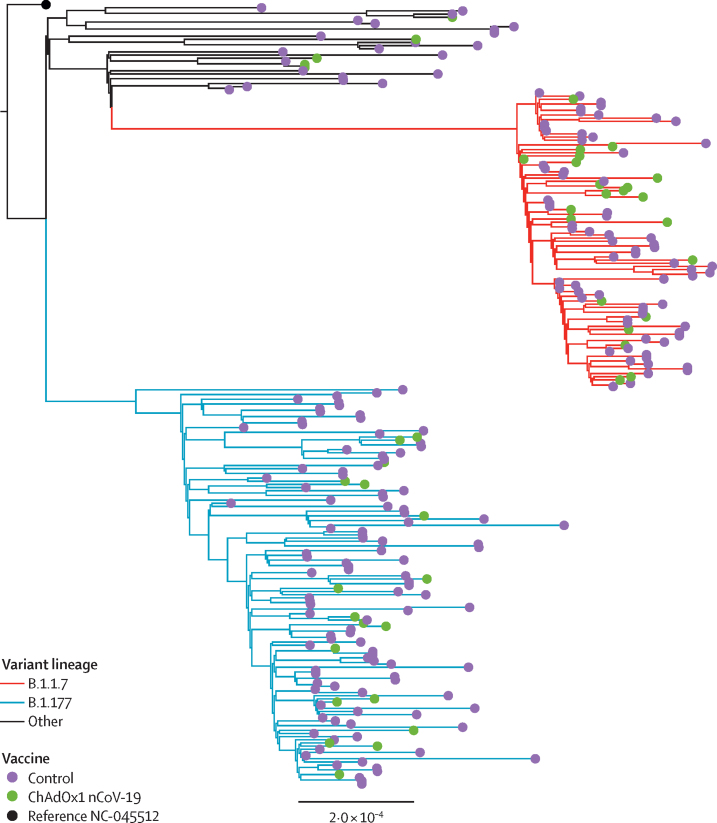
Consensus phylogeny of SARS-CoV-2 genomes identified in this study Clades are coloured by variant lineage and tips are coloured by vaccine allocation. Only genomes with at least 40% coverage are included (n=247). Lineages were assigned by Pangolin version 2.1.7 (lineages version 2021–02–12).

**Figure 3:**
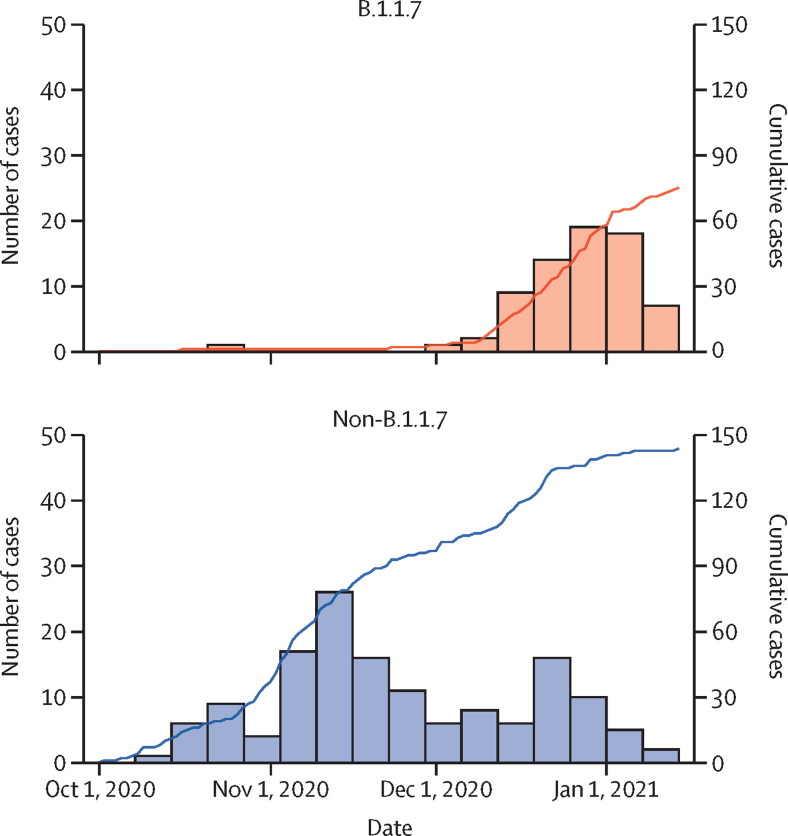
Weekly and cumulative number of B.1.1.7 and non-B.1.1.7 isolates identified in the UK trial between Oct 1, 2020, and Jan 14, 2021 Bars show overall weekly case counts (left axis) and lines show cumulative case counts (right axis).

For primary symptomatic cases for which sequence data were available, 52 (35%) of 147 were due to the B.1.1.7 variant and 95 (65%) were caused by non-B.1.1.7 lineages. Asymptomatic infections or those with unknown symptoms were similarly distributed, with 19 (36%) of 53 infections due to the B.1.1.7 variant and 34 (64%) due to non-B.1.1.7 lineages. The majority of cases of non-B.1.1.7 lineage were caused by B.1.177 (figure 2). Cases of B.1.1.7 first arose in late November, 2020, in trial sites in London and formed an increasing proportion of positive swabs during December, 2020, and January, 2021 (figure 3).

Vaccine efficacy of ChAdOx1 nCoV-19 was 70·4% (95% CI 43·6 to 84·5) against symptomatic COVID-19 caused by the B.1.1.7 variant and 81·5% (67·9 to 89·4) against symptomatic COVID-19 caused by non-B.1.1.7 variants. For cases of asymptomatic or unknown symptom infection obtained from weekly self-swabs, vaccine efficacy was higher for non-B.1.1.7 infections (69·7%, 33·0 to 86·3) than for B.1.1.7 (28·9%, −77·1 to 71·4), although fewer cases were available for analysis, so CIs are wide and overlapping. In contrast, no efficacy was seen for asymptomatic or unknown infections that were not sequenced (5·6%, −42·3 to 37·3) or for which no sequence result was obtained (−27·0%, −108·1 to 22·5). Overall efficacy from all cases was 61·7% (36·7 to 76·9) against the B.1.1.7 variant and 77·3% (65·4 to 85·0) against other variants (table). Baseline demographics in individuals with the B.1.1.7 variant were similar to those with non-B.1.1.7 variants, as were their prime-boost intervals (appendix pp 2–4).

**Table:** Vaccine efficacy against B.1.1.7 and non-B.1.1.7 variants

	Cases*	**ChAdOx1 nCoV-19 vaccine (n=4244)**	**Control vaccine (n=4290)**	**ChAdOx1 nCoV-19 vaccine efficacy (95% CI)**
**Primary symptomatic COVID-19**				
B.1.1.7	52 (19%)	12	40	70·4% (43·6 to 84·5)
Other variants	95 (35%)	15	80	81·5% (67·9 to 89·4)
No sequence result†	30 (11%)	5	25	80·2% (48·3 to 92·4)
Not sequenced‡	92 (34%)	27	65	59·1% (36·0 to 73·9)
Total cases	269	59	210	72·3% (63·1 to 79·3)
**Asymptomatic or unknown infection**				
B.1.1.7	19 (9%)	8	11	28·9% (−77·1 to 71·4)
Other variants	34 (16%)	8	26	69·7% (33·0 to 86·3)
No sequence result†	64 (31%)	36	28	−27·0% (−108·1 to 22·5)
Not sequenced‡	92 (44%)	45	47	5·6% (−42·3 to 37·3)
Total cases	209	97	112	14·6% (−12·1 to 34·9)
**Any NAAT positive infection**§				
B.1.1.7	75 (14%)	21	54	61·7% (36·7 to 76·9)
Other variants	144 (28%)	27	117	77·3% (65·4 to 85·0)
No sequence result†	101 (19%)	44	57	23·7% (−13·0 to 48·5)
Not sequenced‡	200 (38%)	81	119	32·9% (11·0 to 49·5)
Total cases	520	173	347	50·9% (41·0 to 59·0)

Data include SD/SD and LD/SD seronegative efficacy cohorts only. NAAT=nucleic acid amplification test. SD=standard dose. LD=low dose.

* Data in this column are n (%) or n.

† No viable sequence obtained or unprocessed due to cycle threshold >30.

‡ Sample did not enter sequencing pipeline, was destroyed, or sequencing results are yet to be obtained.

§ Includes primary symptomatic cases, non-primary symptomatic cases (those with other symptoms such as nausea or diarrhoea; not shown separately), asymptomatic cases, and cases for which symptoms were unknown.

36 (69%) of 52 cases of primary symptomatic COVID-19 caused by the B.1.1.7 variant occurred in participants who received SD/SD vaccines, and 16 (31%) occurred in participants who received LD/SD vaccines. Non-B.1.1.7 variant cases of primary symptomatic COVID-19 were similarly distributed with 61 (64%) of 95 in the SD/SD group and 34 (36%) in the LD/SD group. Vaccine efficacy against the B.1.1.7 variant was 66·7% (95% CI 29·2–84·3) in the SD/SD group and 77·9% (22·5–93·7) in the LD/SD group. For non-B.1.1.7 variant cases, vaccine efficacy was 78·0% (57·7–88·5) in the SD/SD group compared with 87·2% (63·7–95·5) in the LD/SD group. When stratified by SD/SD or LD/SD group, few cases were available for robust comparisons for asymptomatic or unknown infections (appendix p 5).

No participants from the cohorts in this study were hospitalised or died due to COVID-19 disease.

**Figure 4:**
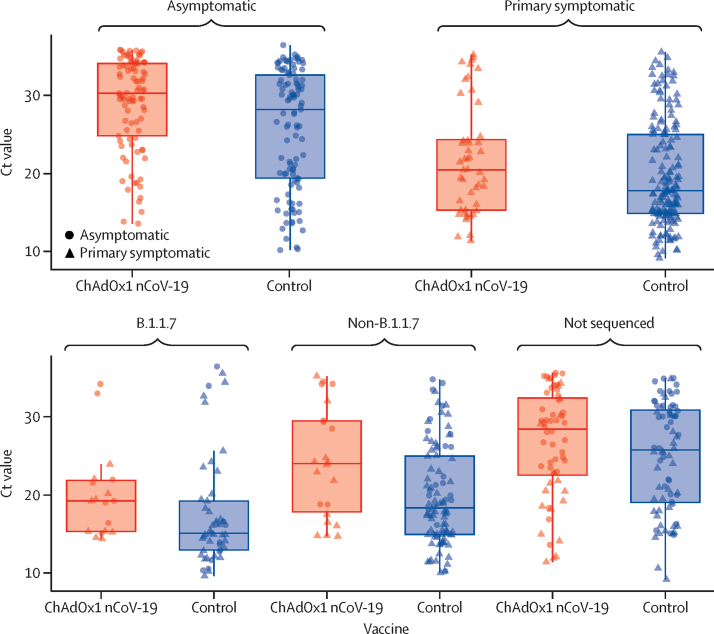
Minimum Ct values across all NAAT positive swabs Ct values from positive NAATs performed at Lighthouse Laboratories using a ThermoFisher TaqPath three-gene assay. Each datapoint represents one participant. For each participant, the minimum of the Ct value for the N gene and ORF1ab gene was taken for each NAAT positive swab, and the minimum across all swabs for the same participant was calculated as a proxy for maximum viral load. Low Ct values are associated with a higher viral load. The midlines of the boxes show medians and the outer bounds of the boxes show IQRs. Error bars show the most extreme point within 1.5 × IQR above or below the 75th or 25th percentile. Ct=cycle threshold. NAAT=nucleic acid amplification test.

**Figure 5:**
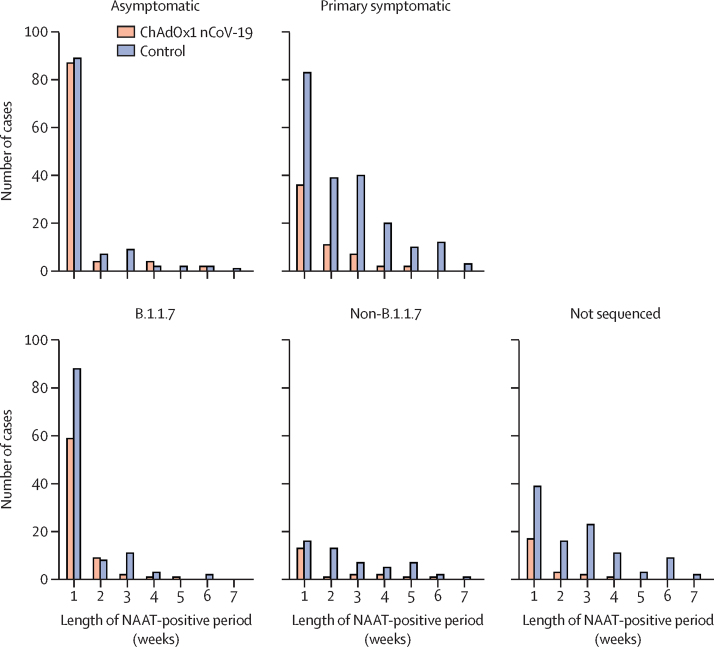
Length of the NAAT-positive period per participant Three primary symptomatic participants who received ChAdOx1 nCoV-19 remained NAAT positive for 8, 9, and 11 weeks, respectively, and one primary symptomatic participant who received the control vaccine remained NAAT positive for 11 weeks; these cases are not shown in the figure. NAAT=nucleic acid amplification test.

Minimum Ct values (an inverse proxy for peak viral load) from Lighthouse Laboratory swabs in participants who received ChAdOx1 nCoV-19 (median 28·8, IQR 20·5–33·5) were higher than in those who received the control vaccine (20·2, 15·5–29·6, p<0·0001; appendix p 6) and participants were NAAT positive for a shorter period of time (p<0·0001; appendix p 7). Primary symptomatic cases had lower Ct values than asymptomatic or unknown symptom cases (median 18·2 [15·0–25·0] *vs* 29·7 [21·6–33·5], primary *vs* asymptomatic, p<0·0001; figure 4) and were NAAT positive for a longer period of time (p<0·0001; figure 5). Almost all (169 [81%] of 209) asymptomatic participants returned only one positive swab. However, primary symptomatic cases remained NAAT positive for longer, with only 56 (21%) of 269 returning only one positive swab. Primary symptomatic participants who received ChAdOx1 nCoV-19 had a shorter NAAT-positive period (median 1·0 week, IQR 1·0–2·0) than those who received the control vaccine (2·0 weeks, 1·0–3·0). The number of weeks positive in B.1.1.7 variant cases was not different to the non-B.1.1.7 variant cases (p=0·85; appendix p 7).

**Figure 6:**
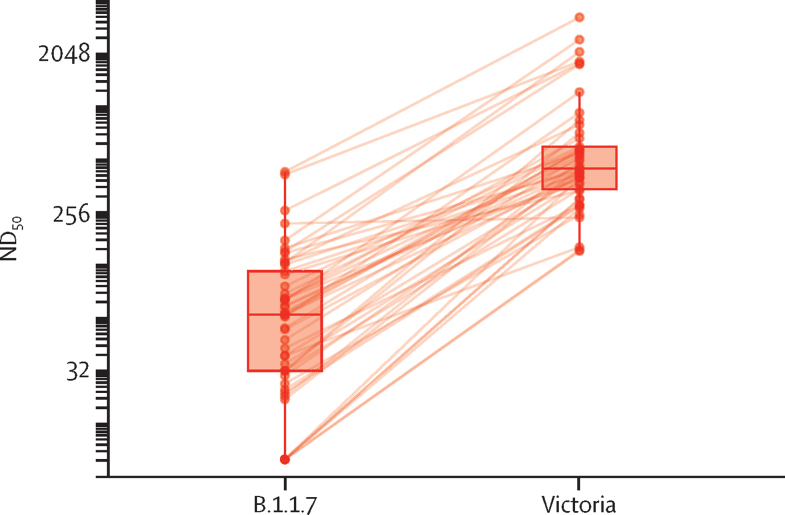
Live-virus microneutralisation antibody titres of sera against B.1.1.7 and a canonical non-B.1.1.7 strain (Victoria) The geometric mean titre is 58 (95% CI 44–77) for B.1.1.7 and 517 (424–631) for Victoria. The geometric mean ratio (Victoria *vs* B.1.1.7) is 8·9 (95% CI 7·2–11·0). The midlines of the boxes show medians and the outer bounds of the boxes show IQRs. Error bars show the most extreme point within 1·5 × IQR above or below the 75th or 25th percentile. Lines connect samples from the same participant collected at the same trial timepoint (n=49). ND_50_=titre at which 50% virus neutralisation is achieved.

In a live-virus neutralisation assay (n=49), neutralising titres of ChAdOx1 nCoV-19 recipient sera were nine times lower against the B.1.1.7 lineage than against the Victoria lineage (geometric mean ratio 8·9, 95% CI 7·2–11·0; figure 6).

Using an ELISA that detected total IgG against trimeric spike protein, we observed that participants who had previously received a different ChAdOx1 vector vaccine had similar anti-spike antibody titres at all timepoints after vaccination to ChAdOx1-naive individuals (appendix pp 9–10).

## Discussion

Our findings show that while laboratory neutralising antibody titres generated by vaccination with ChAdOx1 nCoV-19 vaccine are lower for the B.1.1.7 lineage, clinical vaccine efficacy against symptomatic COVID-19 was observed for the B.1.1.7 variant at 70·4%, with a lower bound of 43·6% for the 95% CI. These findings suggest either that lower neutralising antibody titres are sufficient to provide protection or that other mechanisms of immunity could be responsible for protection from disease in vaccinated individuals. The efficacy against this new variant is an important finding for regions where B.1.1.7 is now the dominant variant and vaccine programmes are already underway. However, it should be noted that further mutations in spike protein observed in other novel lineages^7^, ^8^ appear to be driven by escape of the virus from the neutralising activity of public antibodies,^40^, ^41^ indicating that prevention of transmission might be temporary as the virus adapts to natural or vaccine-induced immunity.

Paired comparison of neutralising antibody activity of sera from ChAdOx1 nCoV-19 vaccinees showed reduced activity against the B.1.1.7 lineage compared with a canonical non-B.1.1.7 lineage (Victoria). The Victoria lineage is phylogenetically similar to the original Wuhan outbreak lineage and has only a single mutation in spike protein (S247R; Ser247Arg). Vaccine sera from the Pfizer–BioNTech BNT162b2 mRNA recipients showed no change in neutralising activity using pseudovirus assays comparing a vesicular stomatitis virus (VSV) expressing B.1.1.7 or ancestral Wuhan lineage spike protein.^26^ Sera from recipients of the Moderna mRNA-1273 vaccine showed similar neutralising activity against VSV and lentivirus pseudoviruses expressing full-length spike protein from either B.1.1.7 or a Wuhan lineage,^42^ and this finding was also seen in a live neutralisation assay.^43^ However, a further live coronavirus neutralisation assay showed that sera from BNT162b2 and ChAdOx1 nCoV-19 vaccinees had a 2·0–3·3 times reduction in neutralisation activity against B.1.1.7 compared with the Victoria lineage,^44^ in keeping with our findings. In the absence of a standardised method, these apparently different results with neutralising antibody assays using pseudoviruses and live virus highlight the need for caution in interpretation of viral neutralisation data. Further work is needed to compare in parallel sera from individuals immunised with different vaccines within the same in-vitro assay.

Despite the large reduction in measured live neutralising activity against B.1.1.7 virus, the vaccine provided strong protection against B.1.1.7 variant disease, with the lower bound of the 95% CI above the 30% threshold recommended by WHO.^45^ In line with previously published data,^3^, ^46^ protection was also shown against the non-B.1.1.7 strains, the majority of which were due to the B.1.177 lineage.

The number of samples available for analysis was not sufficient to power a study to determine whether there was a small difference in efficacy between the B.1.1.7 and non-B.1.1.7 variants; therefore, we cannot rule out an 11% reduction in efficacy. A similar finding of a 10% reduction in efficacy against the B.1.1.7 variant was reported for the adjuvanted spike protein-based vaccine, Novavax NVX-CoV2373, from a smaller number of sequences.^4^ This vaccine is also based on the Wu-1 reference spike gene sequence. If these results are confirmed on formal analysis, they will support our finding that immune responses elicited by ancestral spike protein offer a high degree of cross-protection against the B.1.1.7 lineage. This suggestion is encouraging and would indicate that currently deployed vaccines that are also based on early circulating viral lineages (including mRNA and inactivated vaccines) are also likely to be protective against B.1.1.7. ChAdOx1 nCoV-19 has been deployed in the UK national vaccination programme since Jan 4, 2021, during which time B.1.1.7 has been the dominant circulating lineage.^13^ Early national vaccine effectiveness data has shown that a single dose of ChAdOx1 nCoV-19 confers protection from both symptomatic disease and COVID-19-related hospitalisation,^47^ consistent with the findings presented here. No participants in this study had evidence of the novel E484K (Glu484Lys) spike mutation first described in the B1.351 lineage identified in South Africa. The E484K mutation appears to result in a lower neutralising ability of mRNA vaccine sera.^48^ Preliminary data suggest that, in common with other ancestral spike-based vaccines, ChAdOx1 nCov-19 might have a lower clinical efficacy against upper respiratory infection caused by viral lineages bearing this mutation.^49^ Further work is ongoing to ascertain the impact of E484K on the neutralising activity and efficacy afforded by ChAdOx1 nCov-19.

Preclinical COVID-19 vaccine studies suggest that the development of neutralising antibody is associated with protection from subsequent challenge with live virus.^50^ In the absence of a formal correlate of protection, vaccine developers have therefore measured neutralising antibody titres post vaccination as a surrogate of likely efficacy. Our findings that ChAdOx1 nCoV-19 offers protection against the B.1.1.7 lineage could mean that a lower absolute level of neutralising antibody is needed to prevent symptomatic infection.

Different immune mechanisms could also be responsible for the protection afforded by ChAdOx1 nCoV-19. Antibody-dependent complement deposition and antibody-dependent natural killer cell activation are associated with protection from challenge in animal models.^51^ We have previously shown that ChAdOx1 nCoV-19 elicits robust antibody-dependent monocyte phagocytosis, antibody-dependent neutrophil phagocytosis activity, and antibody-dependent natural killer cell activation after two doses of vaccine,^29^ at similar levels to those seen in convalescent sera. Antibodies mediating these functions could have complementarity determining regions that recognise and bind to alternative conformational epitopes on spike protein to those bound by neutralising antibodies, explaining the observed clinical efficacy against B.1.1.7.

T-cell responses are associated with recovery from clinical COVID-19 disease^52^ and we have previously reported that ChAdOx1 nCoV-19 generates spike-specific T cells that peak at 14 days after priming vaccination.^34^ We have previously shown that the peptide pools most frequently recognised by vaccine-elicited T cells span amino acids 311–430 and 101–200 of the S1 domain, which are unaffected by the mutations seen in B.1.1.7. These findings support the suggestion that cellular immune responses to ChAdOx1 nCoV-19 are sustained against the new variant, independently of neutralising activity. Further evaluation of the cross-reactivity of vaccine-derived T cell clones to B.1.1.7 spike peptides is underway.

Vaccine efficacy against asymptomatic infection using sequenced swabs was high (70%) for non-B.1.1.7 variants. It was higher than our previously published estimate of 27%, which was based on NAAT positivity alone,^3^ and substantially higher than the efficacy seen in the asymptomatic cases that have not been sequenced (6%), or in those for whom no result from sequencing could be obtained (−27%). It is possible that some of the specimens which could not be sequenced were false-positive samples generated by low-level plate contamination or amplicon contamination in the NAAT assay. These false positives would fall into the category with −27% efficacy, along with true-positive specimens with very low absolute viral loads or degraded RNA. The contrasting results for efficacy against asymptomatic disease when using sequencing as a confirmation of the presence of virus in the specimen are notable and point to the potential for false-positive NAAT results to alter efficacy estimates. In our study, more than 200 000 nose and throat swabs from participants self-swabbing at home have been tested by NAAT. The false-positive rate within Lighthouse Laboratories used for the community testing is unknown, although early estimates based on other historical RNA viral RT-PCR testing suggested rates of 0·8–4·0%.^53^ Assuming a low false-positive rate of 0·1%, this equates to 200 false-positive swabs, which could skew an attempt to estimate vaccine efficacy. The higher estimate of efficacy against asymptomatic infection in this study from sequenced swabs is less likely to be affected by false-positive results and therefore might be more reliable. However, inflated efficacy against asymptomatic infection could also be caused by lower viral loads in true-positive samples from vaccine recipients, making sequencing more difficult for these samples, thereby lowering the counts in the vaccine group of the study and inflating vaccine efficacy for asymptomatic infections. This bias would be unlikely to affect primary symptomatic cases, for which viral loads are higher.

The presence of viral RNA detected by NAAT in a diagnostic swab might not represent transmissible live virus. Determining exact Ct thresholds associated with infectivity is complicated by different RT-PCR platforms, specimen types, and gene targets, but the probability of recovery of live virus appears reduced from samples with high Ct values.^54^, ^55^, ^56^ Previous studies have shown no difference in viral load between symptomatic and asymptomatic individuals.^54^, ^57^ In our study, individuals who did not report symptoms had lower viral loads than symptomatic individuals and were NAAT positive for a shorter period of time. This finding is consistent with published data that asymptomatic individuals might be responsible for fewer secondary transmissions than symptomatic individuals.^58^ The viral load among those with a NAAT-positive swab in the ChAdOx1 nCoV-19 vaccinated group was statistically significantly lower than among those who were in the control group. Taken with our recent analysis,^46^ which showed a 64% reduction in any NAAT-positive result after a single dose of ChAdOx1 nCoV-19, our findings suggest that even those vaccinees with a NAAT-positive swab could be less likely to transmit the virus than an unvaccinated NAAT-positive individual. These observations provide strong support for mass vaccination as a tool to control pandemic coronavirus, when the vaccine is reasonably well matched to the circulating variant.

Since December, 2020, regulatory authorities around the world have approved emergency use of several COVID-19 vaccines as part of the strategy to combat COVID-19 disease. As an increasing proportion of the population is vaccinated, selection of mutations that allow immune evasion may occur, requiring revaccination with antigens derived from the new lineages. Concerns exist that repeated doses of adenoviral vaccine vectors could generate anti-vector immunity, which could impede responses to the pathogen transgene. We have previously shown that anti-ChAdOx1 vector antibodies do not affect anti-spike antibody responses or spike-specific T-cell responses.^34^ In this study, we showed that individuals who had received a previous ChAdOx1-vectored vaccine for a non-spike transgene at least 1 year before receipt of ChAdOx1 nCoV-19 had similar binding antibody responses to spike protein to ChAdOx1-naive individuals. However, the impact on transgene responses of, for example, an annual vaccination with the same adenoviral vector remains uncertain and heterologous prime-boost strategies incorporating different vaccine platforms might be required.

A limitation of this study is that sequences could not be obtained from all positive swab samples due to logistical constraints in laboratories processing multiple clinical samples during the COVID-19 pandemic. The sample size for this analysis was determined by sequence availability. However, the emergence of the B.1.1.7 variant as dominant lineage within the UK coupled with the roll-out of the ChAdOx1 nCoV-19 vaccine justified an exploratory analysis. There were no cases of hospitalisation or death in these trial participants, so we cannot comment on the efficacy of the vaccine on these endpoints.

Although human error in the receiving and sequencing laboratories and missteps in quality control of data matching between laboratories could have affected data veracity, in most cases multiple swabs were obtained from the same individual over a period of weeks, allowing corroboration of in-host minor variants, and providing certainty of sequence linkage to a given participant. The sequences obtained for this study were generated by multiple sequencing laboratories affiliated with the COVID-19 Genomics UK Consortium, using local infrastructure for library preparation and sequencing, with independent quality control procedures performed at each sequencing site and centrally within COVID-19 Genomics UK. The use of heterogeneous sequencing data from high-throughput processes warrants some caution in the interpretation of consensus genomes. Only genomes that had no evidence of contamination were included in this analysis, with sequences showing evidence of mixed variant calls at multiple lineage-defining sites being manually excluded. Furthermore, samples with a high Ct value (>30) were not routinely sequenced by several COVID-19 Genomics UK laboratories, thus limiting our ability to assess the lineage of low viral load specimens, which were over-represented in asymptomatic participants.

The emergence of new lineages of SARS-CoV-2 due to viral mutation and immune and fitness selection is inevitable. Here, we show that the ChAdOx1 nCoV-19 vaccine provides protection against symptomatic disease caused by the novel B.1.1.7 lineage. Vaccination with ChAdOx1 nCoV-19 also results in a reduction in the duration of shedding and viral load, which might reduce transmission of disease, supporting the ongoing use of this vaccine to protect populations at risk of disease.

## Data sharing

Anonymised participant data will be made available when the trial is complete, upon request directed to the corresponding author. Proposals will be reviewed and approved by the sponsor, investigator, and collaborators on the basis of scientific merit. After approval of a proposal, data can be shared through a secure online platform after signing a data access agreement. All data will be made available for a minimum of 5 years from the end of the trial.

## Declaration of interests

Oxford University has entered into a partnership with AstraZeneca for further development of ChAdOx1 nCoV-19. AstraZeneca reviewed the data from the study and the final manuscript before submission but the authors retained editorial control. SCG is cofounder of Vaccitech (collaborators in the early development of this vaccine candidate) and is named as an inventor on a patent covering use of ChAdOx1-vectored vaccines (PCT/GB2012/000467) and a patent application covering this SARS-CoV-2 vaccine. TL is named as an inventor on a patent application covering this SARS-CoV-2 vaccine and was a consultant to Vaccitech. PMF is a consultant to Vaccitech. AJP is chair of the UK Department of Health and Social Care Joint Committee on Vaccination and Immunisation but does not participate in policy advice on coronavirus vaccines, and is a member of the WHO Strategic Advisory Group of Experts. AJP and SNF are NIHR senior investigators. AVSH is a cofounder of and consultant to Vaccitech and is named as an inventor on a patent covering design and use of ChAdOx1-vectored vaccines (PCT/GB2012/000467). MDS reports grants from Janssen, GlaxoSmithKline, Medimmune, Novavax, and MCM Vaccine, and grants and non-financial support from Pfizer outside of the submitted work. CMG reports personal fees from the Duke Human Vaccine Institute outside of the submitted work. ADD reports grants and personal fees from AstraZeneca outside of the submitted work. SNF reports grants from Janssen and Valneva outside of the submitted work. TLV and JV are employees of AstraZeneca. All other authors declare no competing interests.
