# Supplementary material for: Efficacy of ChAdOx1 nCoV-19 (AZD1222) vaccine against SARS-CoV-2 variant of concern 202012/01 (B.1.1.7): an exploratory analysis of a randomised controlled trial
Source: Lancet. 2021 Apr 10;397(10282):1351–62. doi: 10.1016/S0140-6736(21)00628-0 (PMC8009612; doi:10.1016/S0140-6736(21)00628-0)
Supplement: Supplementary appendix [file mmc1.pdf]

THE LANCET

Supplementary appendix

This appendix formed part of the original submission and has been peer reviewed. We post it as supplied by the authors.

Supplement to: Emary KRW, Golubchik T, Aley PK, et al. Efficacy of ChAdOx1 nCoV-19 (AZD1222) vaccine against SARS-CoV-2 variant of concern 202012/01 (B.1.1.7): an exploratory analysis of a randomised controlled trial. *Lancet* 2021; published online March 30. http://dx.doi.org/10.1016/S0140-6736(21)00628-0.

Supplementary Material

Table of Contents

TABLE S1A DEMOGRAPHICS IN THE SD/SD AND LD/SD PRIMARY EFFICACY COHORT (CHADOX1 NCOV-19 AND CONTROL RECIPIENTS POOLED) 2
TABLE S1B DEMOGRAPHICS IN THE SD/SD AND LD/SD PRIMARY EFFICACY COHORT, RECIPIENTS OF CHADOX1 NCOV-19 ONLY 3
TABLE S1C DEMOGRAPHICS IN THE SD/SD AND LD/SD PRIMARY EFFICACY COHORT, RECIPIENTS OF CONTROL (MENACWY) ONLY 4
TABLE S2 VACCINE EFFICACY AGAINST B.1.1.7 AND NON- B.1.1.7 LINEAGES FOR SD/SD AND LD/SD SERONEGATIVE PARTICIPANTS 5
TABLE S3 SUMMARY STATISTICS FOR CT VALUES FROM LIGHTHOUSE LABORATORY NAAT ASSAYS 6
TABLE S4 SUMMARY STATISTICS OF THE NUMBER OF WEEKS OF THE NAAT-POSITIVE PERIOD PER PARTICIPANT 7
TABLE S5 PRIOR CHADOX1 RECIPIENTS 8
FIGURE S1. STUDY PROFILE INDICATING FLOW OF PARTICIPANTS IN THE COV002 TRIAL 9
FIGURE S2. EFFECT OF RECEIPT OF A DIFFERENT PRIOR CHADOX1 VACCINE ON ANTI-SPIKE IGG TITRES BY STANDARDISED ELISA 10
TABLE S6 SUMMARY STATISTICS FOR ANTI-SPIKE IGG BY STANDARDISED ELISA IN PARTICIPANTS WITH AND WITHOUT PRIOR CHADOX1 VACCINATION 11
STATISTICAL MODELS 12
SEQUENCING METHODS 12
THE OXFORD TRIAL GROUP 13
THE COVID-19 GENOMICS UK (COG-UK) CONSORTIUM 25
AMPHEUS PROJECT........ 33
ACKNOWLEDGEMENTS........ 33

Table S1A Demographics in the SD/SD and LD/SD primary efficacy cohort (ChAdOx1 nCoV-19 and control recipients pooled)

Demographics	Primary efficacy cohort (n=8534)	NAAT+ cases (n=520)	B.1.1.7 (n=75)	Non-B.1.1.7 (n=144)	No result (n=101)	Not sequenced (n=200)	p-value B.1.1.7 vs non-B.1.1.7*
Age							
18-55 years	6636 (77.8%)	459 (88.3%)	65 (86.7%)	122 (84.7%)	87 (86.1%)	185 (92.5%)	0.912
56-69 years	955 (11.2%)	33 (6.3%)	6 (8.0%)	16 (11.1%)	5 (5.0%)	6 (3.0%)	
≥70 years	943 (11.0%)	28 (5.4%)	4 (5.3%)	6 (4.2%)	9 (8.9%)	9 (4.5%)	
missing	0 (0.0%)	0 (0.0%)	0 (0.0%)	0 (0.0%)	0 (0.0%)	0 (0.0%)	
Sex (female) n%	5065 (59.4%)	326 (62.7%)	44 (58.7%)	89 (61.8%)	67 (66.3%)	126 (63.0%)	0.652
BMI (median, IQR) kg/m^2	25.4 [22.9, 28.9]	25.6 [23.2, 29.7]	25.2 [22.6, 28.0]	26.1 [23.2, 30.1]	25.3 [22.9, 27.9]	25.6 [23.4, 30.3]	0.097
Ethnicity							
White	7863 (92.1%)	484 (93.1%)	70 (93.3%)	136 (94.4%)	96 (95.0%)	182 (91.0%)	0.849
Black	40 (0.5%)	2 (0.4%)	0 (0.0%)	0 (0.0%)	0 (0.0%)	2 (1.0%)	
Asian	424 (5.0%)	29 (5.6%)	4 (5.3%)	6 (4.2%)	4 (4.0%)	15 (7.5%)	
Mixed	139 (1.6%)	3 (0.6%)	1 (1.3%)	1 (0.7%)	1 (1.0%)	0 (0.0%)	
Other	68 (0.8%)	2 (0.4%)	0 (0.0%)	1 (0.7%)	0 (0.0%)	1 (0.5%)	
missing	0 (0.0%)	0 (0.0%)	0 (0.0%)	0 (0.0%)	0 (0.0%)	0 (0.0%)	
Health and social care setting workers n%	5623 (65.9%)	378 (72.7%)	48 (64.0%)	109 (75.7%)	68 (67.3%)	153 (76.5%)	0.068
Co-morbidities							
Cardiovascular disease	1029 (12.1%)	54 (10.4%)	5 (6.7%)	18 (12.5%)	8 (7.9%)	23 (11.5%)	0.181
Respiratory disease	1042 (12.2%)	61 (11.7%)	10 (13.3%)	23 (16.0%)	10 (9.9%)	18 (9.0%)	0.604
Diabetes	185 (2.2%)	10 (1.9%)	1 (1.3%)	4 (2.8%)	0 (0.0%)	5 (2.5%)	0.663
Prime-boost interval							
<6 weeks	1742 (20.4%)	63 (12.1%)	10 (13.3%)	20 (13.9%)	16 (15.8%)	17 (8.5%)	0.540
6-8 weeks	1045 (12.2%)	69 (13.3%)	5 (6.7%)	16 (11.1%)	19 (18.8%)	29 (14.5%)	
9-11 weeks	2517 (29.5%)	170 (32.7%)	34 (45.3%)	37 (25.7%)	25 (24.8%)	74 (37.0%)	
≥12 weeks	3230 (37.8%)	218 (41.9%)	26 (34.7%)	71 (49.3%)	41 (40.6%)	80 (40.0%)	

*p-values from Chi-squared and Fisher Exact tests, Wilcoxon Rank Sum tests (BMI) and Cochran-Armitage tests (ordinal age groups and prime-boost intervals), testing for associations between the corresponding variable and B.1.1.7 vs Non-B.1.1.7 variants.

Table S1B Demographics in the SD/SD and LD/SD primary efficacy cohort, recipients of ChAdOx1 nCoV-19 only

Demographics	Primary efficacy cohort (n=4244)	NAAT+ cases (n=173)	B.1.1.7 (n=21)	Non-B.1.1.7 (n=27)	No result (n=44)	Not sequenced (n=81)
Age						
18-55 years	3300 (77.8%)	150 (86.7%)	18 (85.7%)	23 (85.2%)	38 (86.4%)	71 (87.7%)
56-69 years	476 (11.2%)	10 (5.8%)	0 (0.0%)	4 (14.8%)	3 (6.8%)	3 (3.7%)
≥70 years	468 (11.0%)	13 (7.5%)	3 (14.3%)	0 (0.0%)	3 (6.8%)	7 (8.6%)
missing	0 (0.0%)	0 (0.0%)	0 (0.0%)	0 (0.0%)	0 (0.0%)	0 (0.0%)
Sex (female) n%	2485 (58.6%)	110 (63.6%)	10 (47.6%)	15 (55.6%)	31 (70.5%)	54 (66.7%)
BMI (median, IQR) kg/m^2	25.4 [23.0, 28.8]	25.6 [23.2, 30.0]	25.4 [23.0, 27.0]	26.7 [23.1, 30.9]	25.6 [22.9, 28.0]	26.1 [23.5, 30.9]
Ethnicity						
White	3894 (91.8%)	163 (94.2%)	20 (95.2%)	27 (100.0%)	41 (93.2%)	75 (92.6%)
Black	23 (0.5%)	1 (0.6%)	0 (0.0%)	0 (0.0%)	0 (0.0%)	1 (1.2%)
Asian	222 (5.2%)	8 (4.6%)	0 (0.0%)	0 (0.0%)	3 (6.8%)	5 (6.2%)
Mixed	72 (1.7%)	1 (0.6%)	1 (4.8%)	0 (0.0%)	0 (0.0%)	0 (0.0%)
Other	33 (0.8%)	0 (0.0%)	0 (0.0%)	0 (0.0%)	0 (0.0%)	0 (0.0%)
missing	0 (0.0%)	0 (0.0%)	0 (0.0%)	0 (0.0%)	0 (0.0%)	0 (0.0%)
Health and social care setting workers n%	2774 (65.4%)	120 (69.4%)	11 (52.4%)	19 (70.4%)	30 (68.2%)	60 (74.1%)
Co-morbidities						
Cardiovascular disease	514 (12.1%)	27 (15.6%)	2 (9.5%)	8 (29.6%)	5 (11.4%)	12 (14.8%)
Respiratory disease	504 (11.9%)	19 (11.0%)	2 (9.5%)	4 (14.8%)	6 (13.6%)	7 (8.6%)
Diabetes	97 (2.3%)	4 (2.3%)	0 (0.0%)	2 (7.4%)	0 (0.0%)	2 (2.5%)
Prime-boost interval						
<6 weeks	870 (20.5%)	21 (12.1%)	3 (14.3%)	4 (14.8%)	5 (11.4%)	9 (11.1%)
6-8 weeks	548 (12.9%)	41 (23.7%)	3 (14.3%)	5 (18.5%)	14 (31.8%)	19 (23.5%)
9-11 weeks	1222 (28.8%)	47 (27.2%)	9 (42.9%)	6 (22.2%)	9 (20.5%)	23 (28.4%)
≥12 weeks	1604 (37.8%)	64 (37.0%)	6 (28.6%)	12 (44.4%)	16 (36.4%)	30 (37.0%)

Table S1C Demographics in the SD/SD and LD/SD primary efficacy cohort, recipients of control (MenACWY) only

**Demographics**	**Primary efficacy cohort (n=4290)**	**NAAT+ cases (n=347)**	**B.1.1.7 (n=54)**	**Non-B.1.1.7 (n=117)**	**No result (n=57)**	**Not sequenced (n=119)**
Age						
18-55 years	3336 (77.8%)	309 (89.0%)	47 (87.0%)	99 (84.6%)	49 (86.0%)	114 (95.8%)
56-69 years	479 (11.2%)	23 (6.6%)	6 (11.1%)	12 (10.3%)	2 (3.5%)	3 (2.5%)
≥70 years	475 (11.1%)	15 (4.3%)	1 (1.9%)	6 (5.1%)	6 (10.5%)	2 (1.7%)
missing	0 (0.0%)	0 (0.0%)	0 (0.0%)	0 (0.0%)	0 (0.0%)	0 (0.0%)
Sex (female) n%	2580 (60.1%)	216 (62.2%)	34 (63.0%)	74 (63.2%)	36 (63.2%)	72 (60.5%)
BMI (median, IQR) kg/m^2	25.4 [22.9, 28.9]	25.5 [23.1, 29.5]	24.5 [22.4, 28.2]	26.0 [23.4, 29.8]	25.1 [22.9, 27.9]	25.6 [23.4, 29.1]
Ethnicity						
White	3969 (92.5%)	321 (92.5%)	50 (92.6%)	109 (93.2%)	55 (96.5%)	107 (89.9%)
Black	17 (0.4%)	1 (0.3%)	0 (0.0%)	0 (0.0%)	0 (0.0%)	1 (0.8%)
Asian	202 (4.7%)	21 (6.1%)	4 (7.4%)	6 (5.1%)	1 (1.8%)	10 (8.4%)
Mixed	67 (1.6%)	2 (0.6%)	0 (0.0%)	1 (0.9%)	1 (1.8%)	0 (0.0%)
Other	35 (0.8%)	2 (0.6%)	0 (0.0%)	1 (0.9%)	0 (0.0%)	1 (0.8%)
missing	0 (0.0%)	0 (0.0%)	0 (0.0%)	0 (0.0%)	0 (0.0%)	0 (0.0%)
Health and social care setting workers n%	2849 (66.4%)	258 (74.4%)	37 (68.5%)	90 (76.9%)	38 (66.7%)	93 (78.2%)
Co-morbidities						
Cardiovascular disease	515 (12.0%)	27 (7.8%)	3 (5.6%)	10 (8.5%)	3 (5.3%)	11 (9.2%)
Respiratory disease	538 (12.5%)	42 (12.1%)	8 (14.8%)	19 (16.2%)	4 (7.0%)	11 (9.2%)
Diabetes	88 (2.1%)	6 (1.7%)	1 (1.9%)	2 (1.7%)	0 (0.0%)	3 (2.5%)
Prime-boost interval						
<6 weeks	872 (20.3%)	42 (12.1%)	7 (13.0%)	16 (13.7%)	11 (19.3%)	8 (6.7%)
6-8 weeks	497 (11.6%)	28 (8.1%)	2 (3.7%)	11 (9.4%)	5 (8.8%)	10 (8.4%)
9-11 weeks	1295 (30.2%)	123 (35.4%)	25 (46.3%)	31 (26.5%)	16 (28.1%)	51 (42.9%)
≥12 weeks	1626 (37.9%)	154 (44.4%)	20 (37.0%)	59 (50.4%)	25 (43.9%)	50 (42.0%)

Table S2 Vaccine efficacy against B.1.1.7 and non- B.1.1.7 lineages for SD/SD and LD/SD seronegative participants

Variant		N (%)	ChAdOx1 nCoV-19	Control	VE 95% CI
**Primary Symptomatic COVID-19**					
B.1.1.7	SD/SD	36 (69%)	9/2857	27/2904	66.7% (29.2%, 84.3%)
	LD/SD	16 (31%)	3/1387	13/1386	77.9% (22.5%, 93.7%)
Other variants	SD/SD	61 (64%)	11/2857	50/2904	78.0% (57.7%, 88.5%)
	LD/SD	34 (36%)	4/1387	30/1386	87.2% (63.7%, 95.5%)
No sequence result*	SD/SD	22 (73%)	5/2857	17/2904	70.6% (20.3%, 89.1%)
	LD/SD	8 (27%)	0/1387	8/1386	N/a
Not sequenced**	SD/SD	57 (62%)	18/2857	39/2904	53.8% (19.3%, 73.6%)
	LD/SD	35 (38%)	9/1387	26/1386	66.8% (29.2%, 84.5%)
**Asymptomatic/Unknown infections**					
B.1.1.7	SD/SD	9 (47%)	5/2857	4/2904	-25.0% (-365.2%, 66.4%)
	LD/SD	10 (53%)	3/1387	7/1386	58.9% (-58.6%, 89.4%)
Other variants	SD/SD	23 (68%)	5/2857	18/2904	72.2% (25.2%, 89.7%)
	LD/SD	11 (32%)	3/1387	8/1386	64.1% (-35.4%, 90.5%)
No sequence result*	SD/SD	45 (70%)	27/2857	18/2904	-50.0% (-172.1%, 17.3%)
	LD/SD	19 (30%)	9/1387	10/1386	13.7% (-112.1%, 64.9%)
Not sequenced**	SD/SD	62 (67%)	35/2857	27/2904	-29.6% (-114.3%, 21.6%)
	LD/SD	30 (33%)	10/1387	20/1386	52.1% (-2.4%, 77.6%)
**Any PCR+**					
B.1.1.7	SD/SD	47 (63%)	15/2857	32/2904	53.1% (13.6%, 74.6%)
	LD/SD	28 (37%)	6/1387	22/1386	73.9% (35.7%, 89.4%)
Other variants	SD/SD	94 (65%)	19/2857	75/2904	74.7% (58.1%, 84.7%)
	LD/SD	50 (35%)	8/1387	42/1386	81.7% (61.1%, 91.4%)
No sequence result*	SD/SD	73 (72%)	35/2857	38/2904	7.9% (-45.7%, 41.7%)
	LD/SD	28 (28%)	9/1387	19/1386	54.6% (-0.2%, 79.4%)
Not sequenced**	SD/SD	130 (65%)	60/2857	70/2904	14.3% (-21.1%, 39.3%)
	LD/SD	70 (35%)	21/1387	49/1386	58.9% (31.5%, 75.4%)

Table S3 Summary statistics for Ct values from Lighthouse laboratory NAAT assays

		ChAdOx1 nCoV-19					Control					P value*
**Outcome**	**Variant**	**N**	**Mean**	**Median**	**Q1**	**Q3**	**N**	**Mean**	**Median**	**Q1**	**Q3**	
**Primary**	**B.1.1.7**	10	18.0	17.4	15.3	20.2	38	17.9	15.8	14.1	19.5	
	**Non-B.1.1.7**	15	21.7	22.5	16.1	24.3	71	19.1	17.6	14.9	23.4	
	**No result**	5	28.2	30.7	24.0	33.5	25	22.0	19.6	16.2	29.5	
	**Not sequenced**	19	22.3	20.6	15.0	30.3	35	21.9	20.8	16.1	26.7	
	**All**	**49**	**21.9**	**20.6**	**15.4**	**24.5**	**169**	**19.8**	**17.9**	**15.0**	**25.1**	**0.0726**
**Asymptomatic/ Unknown**	**B.1.1.7**	8	22.6	20.5	17.8	27.5	11	17.3	13.7	11.7	16.7	
	**Non-B.1.1.7**	8	28.5	29.5	23.7	34.2	25	22.5	20.7	18.3	27.9	
	**No result**	36	30.9	32.6	29.9	34.5	28	29.8	32.3	27.2	34.3	
	**Not sequenced**	41	28.7	29.5	24.9	33.2	41	27.5	30.1	22.1	31.6	
	**All**	**93**	**29.0**	**30.3**	**24.9**	**34.1**	**105**	**25.9**	**28.3**	**19.5**	**32.6**	**0.0045**
**B.1.1.7**	**All†**	**18**	**20.1**	**19.3**	**15.4**	**22.0**	**49**	**17.8**	**15.2**	**13.0**	**19.3**	**0.0256**
**Non-B.1.1.7**	**All†**	**23**	**24.2**	**24.1**	**17.6**	**29.6**	**96**	**20.0**	**18.4**	**15.0**	**25.1**	**0.0167**
**All**	**All†**	**142**	**26.6**	**28.8**	**20.5**	**33.5**	**274**	**22.1**	**20.2**	**15.5**	**29.6**	**<0.0001**

*P values from Wilcoxon Rank Sum test comparing ChAdOx1 nCoV-19 with Control. Wilcoxon Rank Sum test: primary symptomatic cases vs asymptomatic cases: p<0.0001, B.1.1.7 cases vs non-B.1.1.7 p=0.0087. † includes only primary symptomatic cases, asymptomatic cases and cases where symptoms were unknown. Non-primary symptomatic cases (those with other symptoms such as nausea or diarrhoea) are excluded.

Table S4 Summary statistics of the number of weeks of the NAAT-positive period per participant

	Arm	No. of positive participants	N(%) returning only one positive swab	Median	Q1	Q2	P value*
**Asymptomatic/Unknown**	ChAdOx1 nCoV-19	97	87 (90)	1.0	1.0	1.0	
	Control	112	82 (73)	1.0	1.0	1.0	
	**Overall**	**209**	**169 (81)**	**1.0**	**1.0**	**1.0**	**0.0484**
**Primary symptomatic**	ChAdOx1 nCoV-19	59	20 (34)	1.0	1.0	2.0	
	Control	210	36 (17)	2.0	1.0	3.0	
	**Overall**	**269**	**56 (21)**	**2.0**	**1.0**	**3.0**	**0.0010**
**Asymptomatic/Unknown/Primary symptomatic†**							
**B.1.1.7**	ChAdOx1 nCoV-19	20	9 (45)	1.0	1.0	3.0	
	Control	51	8 (16)	2.0	1.0	4.0	
	**Overall**	**71**	**17 (24)**	**2.0**	**1.0**	**4.0**	**0.0493**
**Non-B.1.1.7**	ChAdOx1 nCoV-19	23	8 (35)	1.0	1.0	1.5	
	Control	106	19 (18)	2.0	1.0	4.0	
	**Overall**	**129**	**27 (21)**	**2.0**	**1.0**	**3.0**	**0.0006**
**Not sequenced**	ChAdOx1 nCoV-19	72	56 (78)	1.0	1.0	1.0	
	Control	112	64 (57)	1.0	1.0	1.0	
	**Overall**	**184**	**120 (65)**	**1.0**	**1.0**	**1.0**	**0.4506**

*P values from Wilcoxon Rank Sum test comparing ChAdOx1 nCoV-19 with Control. Wilcoxon Rank Sum test: primary symptomatic cases vs asymptomatic cases: p<0.0001, B.1.1.7 cases vs non-B.1.1.7 p=0.8516, ChAdOx1 nCoV-19 vs Control (all Asymptomatic/Unknown/Primary symptomatic) p<0.0001. † includes only primary symptomatic cases, asymptomatic cases and cases where symptoms were unknown. Unknown swabs are included as asymptomatic. Non-primary symptomatic cases (those with other symptoms such as nausea or diarrhoea) are excluded.

Table S5 Prior ChAdox1 recipients

Vaccine Administered	Interval between 1st dose of prior ChadOx1 and ChadOx1-nCov19 (months)	Interval between 2nd dose of prior ChadOx1 and ChadOx1-nCov19 (months)
ChAdOx1 MERS	28	N/A
ChAdOx1 MERS	28	N/A
ChAdOx1 MERS	29	N/A
ChAdOx1 MenB.1	25	13
ChAdOx1 MenB.1	26	N/A
ChAdOx1 MenB.1	13	19
ChAdOx1 MenB.1	26	N/A
ChAdOx1 MenB.1	29	N/A
ChAdOx1 MenB.1	26	N/A
ChAdOx1 MenB.1	26	N/A

ChAdOx1 MERS vaccine contained either $5x10^9$ or $5x10^{10}$ virus particles (vp) of a ChAdox1 vector with a sequence encoding Middle East respiratory syndrome (MERS) coronavirus spike protein. ChAdOx1-MenB.1 vaccine contained $5x10^{10}$ vp of a ChAdOx1 vector with a sequence encoding a meningococcal capsular group B surface antigen.

Figure S1. Study profile indicating flow of participants in the COV002 trial

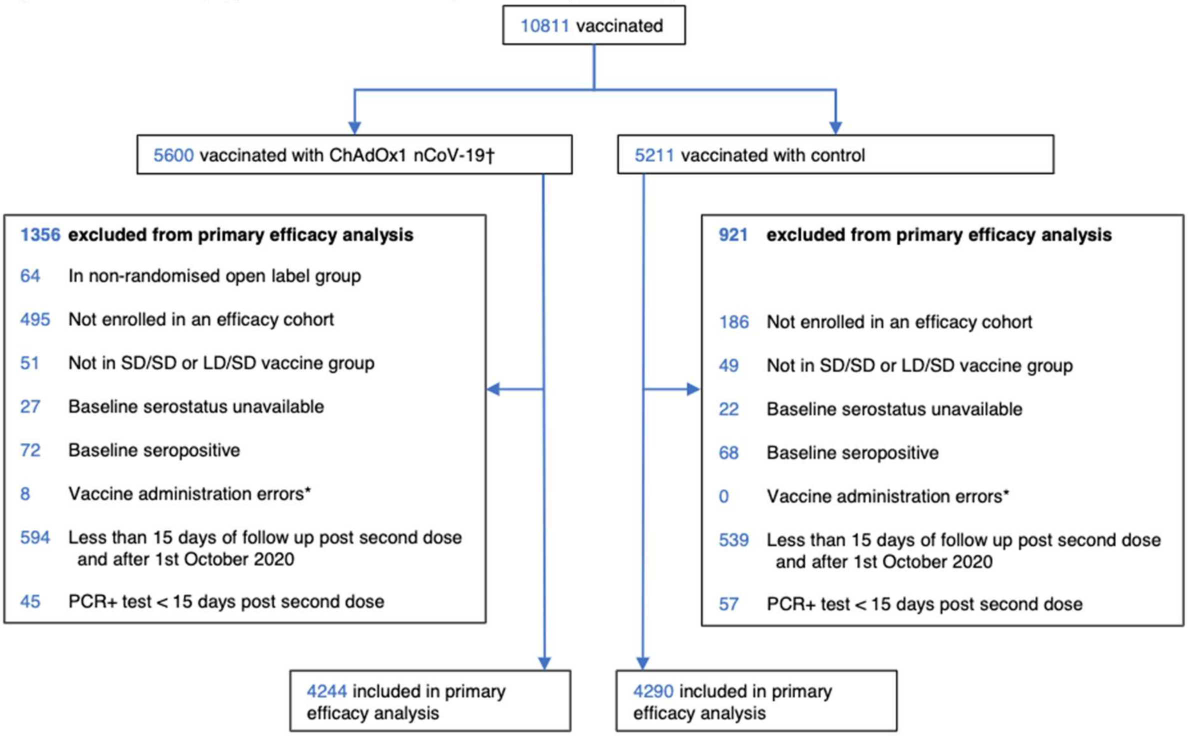

† Includes all participants who received at least a single dose of ChAdOx1 nCoV-19

*Participants who received a different vaccine for their first and second dose. Five participants received a ChAdOx1 nCoV-19 vaccine for their first dose and MenACWY for their second dose, and three received MenACWY for their first dose and ChAdOx1 nCoV-19 for their second dose.

Figure S2. Effect of receipt of a different prior ChAdOx1 vaccine on anti-spike IgG titres by standardised ELISA

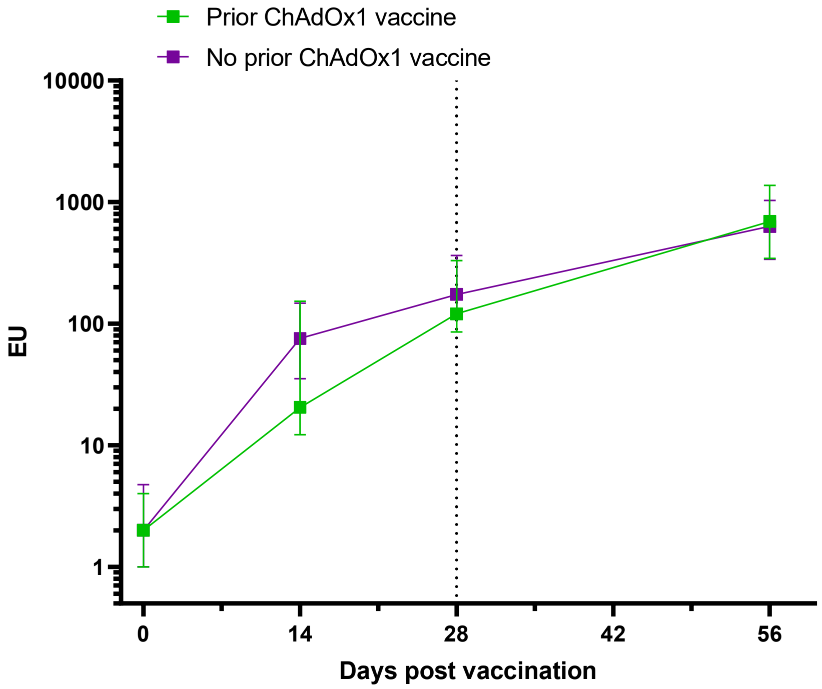

SARS-CoV-2 IgG responses to trimeric spike protein in individuals who had (n=10) and had not (n=48) previously received a ChAdOx1-vectored vaccine. Datapoints are medians with whiskers showing the IQR. All participants received 2 standard doses of ChAdOx1 nCoV-19 at D0 and D28 as indicated by the vertical black line. EU = ELISA units. Data for ChAdOx1 naïve recipients published previously. [34]

Table S6 Summary statistics for anti-spike IgG by standardised ELISA in participants with and without prior ChAdOx1 vaccination

Day	Prior ChAdOx1 Vaccine			No Prior ChAdOx1 Vaccine			
	N	Median [IQR]	GMT (95% CI)	N	Median [IQR]	GMT (95% CI)	p value*
D0	10	2 [1, 4]	1.966 (1.16, 3.333)	48	2 [1, 4.75]	2.532 (1.717, 3.735)	
D14	10	20.5 [12.25, 153]	32.81 (11.36, 94.8)	48	75.5 [35.25, 147.8]	70.51 (47.25, 105.2)	0.133
D28 (B)	10	120.5 [85.5, 331.5]	132.1 (54.55, 319.9)	48	174 [129.5, 364.3]	214.1 (156.3, 293.4)	0.099
D56	10	692.5 [344, 1375]	679.5 (399.4, 1156)	47	631 [338, 1037]	616.5 (478.2, 794.9)	0.684

*Wilcoxon Rank Sum Test. (B) day of boost.

Statistical Models

SAS code for robust Poisson model

```
* model;
proc genmod data=dataset;
      class vaccine_group (param=ref ref='Control') agegroup ldsd subject_id;
      model outcome = group agegroup ldsd / dist=poisson link=log alpha=0.05 offset=log_futime;
      repeated subject= subject_id / type=unstr;
      ods output GEEEmpPEst=temp1;
run;

* calculate rr and confidence interval;
data temp2;
      set temp1;
      rr=exp(Estimate);
      rr_lci=exp(LowerCL);
      rr_uci=exp(UpperCL);
      ve=(1-rr)*100;
      ve_uci=(1-rr_lci)*100;
      ve_lci=(1-rr_uci)*100;
run;
```

Sequencing Methods

Unique dual indexed (UDI) libraries were constructed using the SMARTer Stranded Total RNA-Seq Kit v2—Pico Input Mammalian (Takara Bio USA, California, USA) with no RNA fragmentation. An equal volume of library from each sample was pooled for capture, and size-selected to exclude fragments shorter than 400nt. Target enrichment of SARS-CoV-2 was carried out with a custom xGen Lockdown Probes panel (IDT, Coralville, USA), using the SeqCap EZ Accessory Kits v2 and SeqCap Hybridization and Wash Kit (Roche, Madison, USA) for hybridization of the probes and removal of unbound DNA. Following 12 cycles of PCR for post-capture amplification, the final product was purified using Agencourt AMPure XP (Beckman Coulter, California, USA). Sequencing was performed on the Illumina NovaSeq (Illumina, California, USA) at the Oxford Genomics Centre (OGC), generating 250bp paired-end reads. Each sequencing batch of up to 96 samples included a non-SARS-CoV-2 in-run control (purified *in vitro* transcribed HIV RNA from clone p92BR025.8, obtained from the National Institute for Biological Standards and Control (NIBSC)), as well as positive and negative quantification controls consisting of in vitro transcribed SARS-CoV-2 RNA (Twist Synthetic SARS-CoV-2 RNA Control 1 (MT007544.1), Twist Bioscience) diluted into Universal Human Reference RNA (UHRR) to a final concentration of SARS-CoV-2 RNA of 500,000, 50,000, and 0 copies/reaction. Controls were checked to ensure no evidence of amplification in the negatives and expected RNA quantification consistent with Ct values provided by the testing laboratories.

The Oxford Trial Group

Marites Aban	NIHR Imperial Clinical Research Facility, London, UK
Abdullah Abdullah	Hull University Teaching Hospitals NHS Trust, Hull, UK
Kushala W M Abeysekera	University Hospitals Bristol & Weston NHS Foundation Trust
Jeremy Aboagye	Jenner Institute, Nuffield Department of Medicine, University of Oxford, UK
Matthew Adam	Clinical Infection Research Group, Regional Infectious Diseases Unit, NHS Lothian, UK
Kirsty Adams	NIHR UCLH Clinical Research Facility, London, UK
James P. Adamson	Public Health Wales NHS Trust, Cardiff, UK
Gbadebo Adewetan	London Northwest University Healthcare, Northwick Park Hospital, London, UK
Syed Adlou	Oxford Vaccine Group, Department of Paediatrics, University of Oxford, UK
Khalil Ahmed	Hull University Teaching Hospitals NHS Trust, Hull, UK
Aabidah Ali	Jenner Institute, Nuffield Department of Medicine, University of Oxford, UK
Elizabeth R. Allen	Jenner Institute, Nuffield Department of Medicine, University of Oxford, UK
Lauren Allen	National Infection Service, Public Health England, UK
Nada Al-Muhandis	Hull University Teaching Hospitals NHS Trust, Hull, UK
Rachel Anslow	Oxford Vaccine Group, Department of Paediatrics, University of Oxford, UK
Edward H. Arbe-Barnes	University of Oxford Medical School, Medical Sciences Division, University of Oxford, UK
Markus P. Ariaans	Department of Infection, Immunity and Cardiovascular Disease, University of Sheffield, UK
Gavin Babbage	NIHR Southampton Clinical Research Facility, Southampton, UK
Catherine Bailey	Aneurin Bevan University Health Board, Newport, Wales, UK
Kenneth F. Baker	Department of Infection and Tropical Medicine, Newcastle upon Tyne Hospitals NHS Foundation Trust and Translational and Clinical Research Institute, Immunity and Inflammation Theme, Newcastle University
Megan Baker	Jenner Institute, Nuffield Department of Medicine, University of Oxford, UK
Natalie Baker	National Infection Service, Public Health England, UK
Philip Baker	University of Oxford Medical School, Medical Sciences Division, University of Oxford, UK
Ioana Baleanu	Clinical BioManufacturing Facility, Jenner Institute, University of Oxford, UK
Anna Bara	NIHR Imperial Clinical Research Facility, London, UK
Andrew S. Barr	Department of Infection and Tropical Medicine, Newcastle upon Tyne Hospitals NHS Foundation Trust, UK
Jordan R. Barrett	Jenner Institute, Nuffield Department of Medicine, University of Oxford, UK
Jessica Barrett	London Northwest University Healthcare, Northwick Park Hospital, London, UK
Louise Bates	Oxford Vaccine Group, Department of Paediatrics, University of Oxford, UK
Alexander Batten	Clinical BioManufacturing Facility, Jenner Institute, University of Oxford, UK
Kirsten Beadon	Oxford Vaccine Group, Department of Paediatrics, University of Oxford, UK
Emily Beales	Vaccine Institute, Institute of Infection & Immunity, St. Georges, University of London and St Georges University Hospitals NHS Trust, London, UK
Rebecca Beckley	Oxford Vaccine Group, Department of Paediatrics, University of Oxford, UK
Sandra Belij-Rammerstorfer	Jenner Institute, Nuffield Department of Medicine, University of Oxford, UK
Jonathan Bell	Oxford Vaccine Group, Department of Paediatrics, University of Oxford, UK
Duncan Bellamy	Jenner Institute, Nuffield Department of Medicine, University of Oxford, UK

Sue Belton	The University of Nottingham Health Service, Cripps Health Centre, University Park, Nottingham, UK
Adam Berg	Jenner Institute, Nuffield Department of Medicine, University of Oxford, UK
Eleanor Berrie	Clinical BioManufacturing Facility, Jenner Institute, University of Oxford, UK
Lisa Berry	NIHR Southampton Clinical Research Facility, Southampton, UK
Amy Beveridge	Oxford Vaccine Group, Department of Paediatrics, University of Oxford, UK
Kevin R Bewley	National Infection Service, Public Health England, UK
Inderjeet Bharaj	London Northwest University Healthcare, Northwick Park Hospital, London, UK
Else Margreet Bijker	Oxford Vaccine Group, Department of Paediatrics, University of Oxford, UK
Sarah Birch	Academic Directorate of Communicable Diseases and Specialised Medicine, Sheffield Teaching Hospitals NHS Foundation Trust
Kathryn Birchall	Clinical Research Facility, Sheffield Teaching Hospitals NHS Foundation Trust, UK
Olivia Bird	Vaccine Institute, Institute of Infection & Immunity, St. Georges, University of London and St Georges University Hospitals NHS Trust, London, UK
Karen Bisnauthsing	NIHR BRC at Guy's and St Thomas' NHS Foundation Trust, UK
Mustapha Bittaye	Jenner Institute, Nuffield Department of Medicine, University of Oxford, UK
Luke Blackwell	Oxford Vaccine Group, Department of Paediatrics, University of Oxford, UK
Rachel Blacow	Clinical Research Facility, Queen Elizabeth University Hospital, Glasgow, UK
Heather Bletchly	Oxford Vaccine Group, Department of Paediatrics, University of Oxford, UK
Caitlin L Blundell	Department of Biochemistry, University of Oxford, UK
Susannah R Blundell	Department of Biochemistry, University of Oxford, UK
Pritesh Bodalia	Pharmacy, University College London Hospitals NHS Trust, UK
Emma Bolam	Clinical BioManufacturing Facility, Jenner Institute, University of Oxford, UK
Elena Boland	Clinical BioManufacturing Facility, Jenner Institute, University of Oxford, UK
Nicola Borthwick	Jenner Institute, Nuffield Department of Medicine, University of Oxford, UK
Amy Boyd	Jenner Institute, Nuffield Department of Medicine, University of Oxford, UK
Penny Bradley	Department of Pharmacy, Newcastle upon Tyne Hospitals NHS Foundation Trust, UK
Tanja Brenner	Clinical BioManufacturing Facility, Jenner Institute, University of Oxford, UK
Alice Bridges-Webb	Oxford Vaccine Group, Department of Paediatrics, University of Oxford, UK
Phillip Brown	National Infection Service, Public Health England, UK
Claire Brown	NIHR/Wellcome Trust Birmingham Clinical Research Facility, Birmingham, UK
Charlie Brown-O'Sullivan	Jenner Institute, Nuffield Department of Medicine, University of Oxford, UK
Emily Brunt	National Infection Service, Public Health England, UK
William Budd	NIHR Imperial Clinical Research Facility, London, UK
Jamie Burbage	Oxford Vaccine Group, Department of Paediatrics, University of Oxford, UK
Aileen Burn	Research Directorate, Newcastle upon Tyne Hospitals NHS Foundation Trust, UK
Karen R Buttigieg	National Infection Service, Public Health England, UK
Nicholas Byard	Jenner Institute, Nuffield Department of Medicine, University of Oxford, UK
Ingrid Cabrera Puig	Jenner Institute, Nuffield Department of Medicine, University of Oxford, UK
Anna Calvert	Vaccine Institute, Institute of Infection & Immunity, St. Georges, University of London and St Georges University Hospitals NHS Trust, London, UK
Susana Camara	Oxford Vaccine Group, Department of Paediatrics, University of Oxford, UK
Federica Cappuccini	Jenner Institute, Nuffield Department of Medicine, University of Oxford, UK
Melanie Carr	Oxford Vaccine Group, Department of Paediatrics, University of Oxford, UK

Miles W Carroll	National Infection Service, Public Health England, UK
Andrew Carson-Stevens	Division of Population Medicine, School of Medicine, Cardiff University, UK
Helen R Casey	North Bristol NHS Trust, Bristol, UK
Lucia Carratala Castro	Vaccine Institute, Institute of Infection & Immunity, St. Georges, University of London and St Georges University Hospitals NHS Trust, London, UK
Katrina Cathie	University Hospital Southampton NHS Foundation Trust, UK
Jim Chadwick	National Infection Service, Public Health England, UK
Krishna Chatterjee	NIHR Cambridge Clinical Research Facility, Cambridge, UK
Irina Chelysheva	Oxford Vaccine Group, Department of Paediatrics, University of Oxford, UK
Oliver Chester	Oxford Vaccine Group, Department of Paediatrics, University of Oxford, UK
Sunder Chita	London Northwest University Healthcare, Northwick Park Hospital, London, UK
Jee-Sun Cho	Jenner Institute, Nuffield Department of Medicine, University of Oxford, UK
Liliana Cifuentes	Kennedy Institute of Rheumatology, Nuffield Department of Orthopaedics, The University of Oxford, UK
Elizabeth Clark	Oxford Vaccine Group, Department of Paediatrics, University of Oxford, UK
Matthew Clark	Oxford Vaccine Group, Department of Paediatrics, University of Oxford, UK
Rachel Colin-Jones	Oxford Vaccine Group, Department of Paediatrics, University of Oxford, UK
Hayley Colton	Department of Infection and Tropical Medicine, Sheffield Teaching Hospitals NHS Foundation Trust
Sean Connarty	London Northwest University Healthcare, Northwick Park Hospital, London, UK
Naomi S. Coombes	National Infection Service, Public Health England, UK
Rachel Cooper	Oxford Vaccine Group, Department of Paediatrics, University of Oxford, UK
Tumena Corrah	London Northwest University Healthcare, Northwick Park Hospital, London, UK
Catherine A. Cosgrove	Vaccine Institute, Institute of Infection & Immunity, St. Georges, University of London and St Georges University Hospitals NHS Trust, London, UK
Wendy E. M. Crocker	Jenner Institute, Nuffield Department of Medicine, University of Oxford, UK
Christopher Cunningham	Infectious Diseases Department, Cambridge and Peterborough NHS Foundation Trust, UK
Christina J Cunningham	Oxford Vaccine Group, Department of Paediatrics, University of Oxford, UK
Brad E. Damratoski	Clinical BioManufacturing Facility, Jenner Institute, University of Oxford, UK
Zsofia Danos	Vaccine Institute, Institute of Infection & Immunity, St. Georges, University of London and St Georges University Hospitals NHS Trust, London, UK
Mehreen S Datoo	Jenner Institute, Nuffield Department of Medicine, University of Oxford, UK
Chandrabali Datta	Clinical BioManufacturing Facility, Jenner Institute, University of Oxford, UK
Hannah Davies	Jenner Institute, Nuffield Department of Medicine, University of Oxford, UK
Sophie Davies	Jenner Institute, Nuffield Department of Medicine, University of Oxford, UK
Judith Davies	Oxford Vaccine Group, Department of Paediatrics, University of Oxford, UK
John Davis	Research Directorate, Newcastle upon Tyne Hospitals NHS Foundation Trust, UK
Tesfaye Demissie	Oxford Vaccine Group, Department of Paediatrics, University of Oxford, UK
Amisha Desai	Pharmacy Department, University Hospitals Birmingham NHS Foundation Trust
Claudio Di Maso	Oxford Vaccine Group, Department of Paediatrics, University of Oxford, UK
Tanya Dinesh	Oxford Vaccine Group, Department of Paediatrics, University of Oxford, UK
Francesca R. Donnellan	Jenner Institute, Nuffield Department of Medicine, University of Oxford, UK
Naomi Douglas	Oxford Vaccine Group, Department of Paediatrics, University of Oxford, UK

Charlotte Downing	University of Oxford Medical School, Medical Sciences Division, University of Oxford, UK
Jonathan Drake	University of Oxford Medical School, Medical Sciences Division, University of Oxford, UK
Rachael Drake-Brockman	Oxford Vaccine Group, Department of Paediatrics, University of Oxford, UK
Ruth Elizabeth Drury	Oxford Vaccine Group, Department of Paediatrics, University of Oxford, UK
Andrew D. S. Duncan	Clinical Infection Research Group, NHS Lothian, Edinburgh, UK
Kirstine Eastick	Hull University Teaching Hospitals NHS Trust, Hull, UK
Mandy Edwards	Aneurin Bevan University Health Board, Newport, Wales, UK
Nick J. Edwards	Jenner Institute, Nuffield Department of Medicine, University of Oxford, UK
Frances Edwards	University Hospitals Bristol & Weston NHS Foundation Trust
Omar M. El Muhanna	Clinical BioManufacturing Facility, Jenner Institute, University of Oxford, UK
Sean C. Elias	Jenner Institute, Nuffield Department of Medicine, University of Oxford, UK
Branwen Ellison-Handley	Clinical Research Facility, Sheffield Teaching Hospitals NHS Foundation Trust, UK
Michael J. Elmore	National Infection Service, Public Health England, UK
Marcus Rex English	University of Oxford Medical School, Medical Sciences Division, University of Oxford, UK
Celestine Eshiwe	Hull University Teaching Hospitals NHS Trust, Hull, UK
Mutjaba Ghulam Farooq	Oxford Vaccine Group, Department of Paediatrics, University of Oxford, UK
Sofiya Fedosyuk	Jenner Institute, Nuffield Department of Medicine, University of Oxford, UK
Sally Felle	Oxford Vaccine Group, Department of Paediatrics, University of Oxford, UK
Susie Ferguson	Clinical Infection Research Group, NHS Lothian, Edinburgh, UK
Carla Ferreira Da Silva	Oxford Vaccine Group, Department of Paediatrics, University of Oxford, UK
Richard Fisher	Clinical BioManufacturing Facility, Jenner Institute, University of Oxford, UK
Richard Fitzgerald	Department of Clinical Sciences, Liverpool School of Tropical Medicine, UK
James Fletcher	NIHR Imperial Clinical Research Facility, London, UK
Hazel Fofie	Vaccine Institute, Institute of Infection & Immunity, St. Georges, University of London and St Georges University Hospitals NHS Trust, London, UK
Henry Fok	NIHR BRC at Guy's and St Thomas' NHS Foundation Trust and King's College London British Heart Foundation Centre, School of Cardiovascular Medicine and Sciences, UK
Karen J Ford	Oxford Vaccine Group, Department of Paediatrics, University of Oxford, UK
Jamie Fowler	Jenner Institute, Nuffield Department of Medicine, University of Oxford, UK
Emma Francis	Oxford Vaccine Group, Department of Paediatrics, University of Oxford, UK
Sabrina Fudge	University Hospitals Bristol & Weston NHS Foundation Trust
Julie Furze	Jenner Institute, Nuffield Department of Medicine, University of Oxford, UK
Pablo Galian-Rubio	Clinical BioManufacturing Facility, Jenner Institute, University of Oxford, UK
Harriet Garlant	National Infection Service, Public Health England, UK
Ester German	Department of Clinical Sciences, Liverpool School of Tropical Medicine, UK
Ciaran Gilbride	Jenner Institute, Nuffield Department of Medicine, University of Oxford, UK
Kerry Godwin	National Infection Service, Public Health England, UK
Karishma Gokani	NIHR/Wellcome Trust Birmingham Clinical Research Facility, Birmingham, UK

Jack Goodall	London Northwest University Healthcare, Northwick Park Hospital, London, UK
Jayne Goodwin	Health and Care Research Wales, Cardiff, UK
Giacomo Gorini	Jenner Institute, Nuffield Department of Medicine, University of Oxford, UK
Lara Gracie	Oxford Vaccine Group, Department of Paediatrics, University of Oxford, UK
Nicola Greenwood	Jenner Institute, Nuffield Department of Medicine, University of Oxford, UK
Anishka Gunawardene	London Northwest University Healthcare, Northwick Park Hospital, London, UK
Gaurav Gupta	Jenner Institute, Nuffield Department of Medicine, University of Oxford, UK
Mark Hackett	University Hospitals Bristol & Weston NHS Foundation Trust
Joseph Hamlyn	Oxford Vaccine Group, Department of Paediatrics, University of Oxford, UK
Daniel Hammersley	The University of Nottingham Health Service, Cripps Health Centre, University Park, Nottingham, UK
Aidan T. Hanrath	Department of Infection and Tropical Medicine, Newcastle upon Tyne Hospitals NHS Foundation Trust and Translational and Clinical Research Institute, Immunity and Inflammation Theme, Newcastle University
Brama Hanumunthadu	Oxford Vaccine Group, Department of Paediatrics, University of Oxford, UK
Stephanie A. Harris	Jenner Institute, Nuffield Department of Medicine, University of Oxford, UK
Clair Harris	NIHR BRC at Guy's and St Thomas' NHS Foundation Trust, UK
Thomas D. Harrison	Department of Infection and Tropical Medicine, Sheffield Teaching Hospitals NHS Foundation Trust, UK
Daisy Harrison	Oxford Vaccine Group, Department of Paediatrics, University of Oxford, UK
Thomas C. Hart	Oxford Vaccine Group, Department of Paediatrics, University of Oxford, UK
John Haughney	Clinical Research Facility, Queen Elizabeth University Hospital, Glasgow, UK
Sophia Hawkins	Oxford Vaccine Group, Department of Paediatrics, University of Oxford, UK
Ian Head	University Hospitals Bristol & Weston NHS Foundation Trust
John Aaron Henry	University of Oxford Medical School, Medical Sciences Division, University of Oxford, UK
David B Hettle	Infection Sciences, North Bristol NHS Trust, Bristol, UK
Jennifer Hill	Oxford Vaccine Group, Department of Paediatrics, University of Oxford, UK
Lisa Hitchins	Department of Clinical Sciences, Liverpool School of Tropical Medicine, UK
Gina Hodges	Clinical BioManufacturing Facility, Jenner Institute, University of Oxford, UK
Susanne H. Hodgson	Jenner Institute, Nuffield Department of Medicine, University of Oxford, UK
Mimi M. Hou	Jenner Institute, Nuffield Department of Medicine, University of Oxford, UK
Catherine F. Houlihan	Virology Department, University College London Hospitals NHS Trust, UK and Department of Infection and Immunity, University College London, UK
Ashleigh Howard	Department of Clinical Sciences, Liverpool School of Tropical Medicine, UK
Elizabeth Howe	Oxford Vaccine Group, Department of Paediatrics, University of Oxford, UK
Nicola Howell	Oxford Vaccine Group, Department of Paediatrics, University of Oxford, UK
Holly E. Humphries	National Infection Service, Public Health England, UK
Katrina Hurley	University Hospitals Bristol & Weston NHS Foundation Trust
Catherine Hyams	Academic Respiratory Unit, University of Bristol, Southmead Hospital, Bristol, UK
Sabina Ikram	Vaccine Institute, Institute of Infection & Immunity, St. Georges, University of London and St Georges University Hospitals NHS Trust, London, UK
Alka Ishwarbhai	Jenner Institute, Nuffield Department of Medicine, University of Oxford, UK
Poppy Iveson	University of Oxford Medical School, Medical Sciences Division, University of Oxford, UK
Frederic Jackson	Clinical BioManufacturing Facility, Jenner Institute, University of Oxford, UK

Susan Jackson	Jenner Institute, Nuffield Department of Medicine, University of Oxford, UK
Natasha Jesudason	MRC - University of Glasgow Centre for Virus Research & Department of Infectious Diseases, Queen Elizabeth University Hospital, UK
Carina C. D. Joe	Jenner Institute, Nuffield Department of Medicine, University of Oxford, UK
Christopher Jones	Infection Sciences, North Bristol NHS Trust, Bristol, UK
Kathryn Jones	Jenner Institute, Nuffield Department of Medicine, University of Oxford, UK
Elizabeth Jones	Oxford Vaccine Group, Department of Paediatrics, University of Oxford, UK
Reshma Kailath	Jenner Institute, Nuffield Department of Medicine, University of Oxford, UK
Arnab Kar	London Northwest University Healthcare, Northwick Park Hospital, London, UK
Konstantinos Karampatsas	Vaccine Institute, Institute of Infection & Immunity, St. Georges, University of London and St Georges University Hospitals NHS Trust, London, UK
Mwila Kasanyinga	Oxford Vaccine Group, Department of Paediatrics, University of Oxford, UK
Linda J Kay	Department of Infection, Immunity and Cardiovascular Disease, University of Sheffield
Jade Keen	Oxford Vaccine Group, Department of Paediatrics, University of Oxford, UK
Johanna Kellett Wright	Infection Sciences, North Bristol NHS Trust, Bristol, UK
Elizabeth J. Kelly	AstraZeneca BioPharmaceuticals PLC
Sarah Kelly	Oxford Vaccine Group, Department of Paediatrics, University of Oxford, UK
David Kerr	Oxford Vaccine Group, Department of Paediatrics, University of Oxford, UK
Liaquat Khan	Oxford Vaccine Group, Department of Paediatrics, University of Oxford, UK
Baktash Khozoee	Jenner Institute, Nuffield Department of Medicine, University of Oxford, UK
Ankush Khurana	London Northwest University Healthcare, Northwick Park Hospital, London, UK
Sarah Kidd	University Hospitals Bristol & Weston NHS Foundation Trust
Annabel Killen	University of Oxford Medical School, Medical Sciences Division, University of Oxford, UK
Jasmin Kinch	Oxford Vaccine Group, Department of Paediatrics, University of Oxford, UK
Patrick Kinch	Oxford Vaccine Group, Department of Paediatrics, University of Oxford, UK
Lloyd D. W. King	Jenner Institute, Nuffield Department of Medicine, University of Oxford, UK
Thomas B King	University of Oxford Medical School, Medical Sciences Division, University of Oxford, UK
Lucy Kingham	Jenner Institute, Nuffield Department of Medicine, University of Oxford, UK
Francesca Knapper	University Hospitals Bristol & Weston NHS Foundation Trust
Daniel Knott	National Infection Service, Public Health England, UK
Stanislava Koleva	Oxford Vaccine Group, Department of Paediatrics, University of Oxford, UK
Colin W Larkworthy	Jenner Institute, Nuffield Department of Medicine, University of Oxford, UK
Jessica P J Larwood	University of Oxford Medical School, Medical Sciences Division, University of Oxford, UK
Alison M Lawrie	Jenner Institute, Nuffield Department of Medicine, University of Oxford, UK
Emily A. Lees	Oxford Vaccine Group, Department of Paediatrics, University of Oxford, UK
Alice Lelliott	Oxford Vaccine Group, Department of Paediatrics, University of Oxford, UK
Nana-Marie Lemm	NIHR Imperial Clinical Research Facility, London, UK
Stephanie Leung	National Infection Service, Public Health England, UK
Yuanyuan Li	Jenner Institute, Nuffield Department of Medicine, University of Oxford, UK
Amelia M. Lias	Jenner Institute, Nuffield Department of Medicine, University of Oxford, UK
Konstantinos Liatsikos	Department of Clinical Sciences, Liverpool School of Tropical Medicine and Liverpool University Hospitals NHS Foundation Trust, UK

Aline Linder	Oxford Vaccine Group, Department of Paediatrics, University of Oxford, UK
Samuel Lipworth	Jenner Institute, Nuffield Department of Medicine, University of Oxford, UK
Shuchang Liu	Clinical BioManufacturing Facility, Jenner Institute, University of Oxford, UK
Xinxue Liu	Oxford Vaccine Group, Department of Paediatrics, University of Oxford, UK
Adam Lloyd	Clinical Research Facility, NHS Lothian, Edinburgh, UK
Lisa Loew	Clinical BioManufacturing Facility, Jenner Institute, University of Oxford, UK
Raquel Lopez Ramon	Jenner Institute, Nuffield Department of Medicine, University of Oxford, UK
Jonathan C. MacDonald	Department of Gastroenterology, Queen Elizabeth University Hospital, Glasgow, UK
Gordon MacGregor	Department of Respiratory Medicine, Queen Elizabeth University Hospital, Glasgow, UK
Meera Madhavan	Jenner Institute, Nuffield Department of Medicine, University of Oxford, UK
Rebecca Makinson	Jenner Institute, Nuffield Department of Medicine, University of Oxford, UK
Garry Mallett	University of Oxford Medical School, Medical Sciences Division, University of Oxford, UK
Nicola Manning	University Hospitals Bristol & Weston NHS Foundation Trust
Kushal Mansatta	University of Oxford Medical School, Medical Sciences Division, University of Oxford, UK
Spyridoula Marinou	Oxford Vaccine Group, Department of Paediatrics, University of Oxford, UK
Emma Marlow	Jenner Institute, Nuffield Department of Medicine, University of Oxford, UK
Richard P. Marshall	AstraZeneca BioPharmaceuticals PLC
Julia L. Marshall	Jenner Institute, Nuffield Department of Medicine, University of Oxford, UK
Moncy Mathew	Pharmacy Clinical Trials (Adult), Guy’s and St Thomas NHS Foundation Trust, UK
Olga Mazur	Oxford Vaccine Group, Department of Paediatrics, University of Oxford, UK
Andrea Mazzella	NIHR BRC at Guy's and St Thomas' NHS Foundation Trust, UK
Hugh McCaughan	Laboratory For Bacterial Evolution and Pathogenisis (LBEP), The Roslin Institute, University of Edinburgh, UK
Joanne McEwan	Oxford Vaccine Group, Department of Paediatrics, University of Oxford, UK
Rosa Maeve McGing	Hull University Teaching Hospitals NHS Trust, Hull, UK
Joanna McGlashan	National Infection Service, Public Health England, UK
Lorna McInroy	National Infection Service, Public Health England, UK
Zoe McIntyre	NIHR Cambridge Clinical Research Facility, Cambridge, UK
Tom McLellan	ILD Service, Royal Papworth NHS Foundation Trust, Cambridge, UK
Steve McSwiggan	Clinical Infection Research Group, NHS Lothian, Edinburgh, UK
Savviz Mehdipour	NIHR Imperial Clinical Research Facility, London, UK
Patricia B. Miralhes	Clinical Microbiology and Virology Department, University College London Hospitals NHS Trust, UK
Neginsadat Mirtorabi	University of Oxford Medical School, Medical Sciences Division, University of Oxford, UK
Celia Mitton	Oxford Vaccine Group, Department of Paediatrics, University of Oxford, UK
Fiona Moghaddas	Department of Clinical Immunology, North Bristol NHS Trust, Bristol, UK
Mariya Molai	Hull University Teaching Hospitals NHS Trust, Hull, UK
Ella Morey	Oxford Vaccine Group, Department of Paediatrics, University of Oxford, UK
Róisín Morgans	Clinical BioManufacturing Facility, Jenner Institute, University of Oxford, UK
Susan J. Morris	Clinical BioManufacturing Facility, Jenner Institute, University of Oxford, UK
Sheila Morris	Clinical Infection Research Group, Regional Infectious Diseases Unit, NHS Lothian, UK

Helen C. Morris	NIHR Cambridge Clinical Research Facility, Cambridge, UK
Hazel Morrison	Jenner Institute, Nuffield Department of Medicine, University of Oxford, UK
Franca Morselli	NIHR BRC at Guy's and St Thomas' NHS Foundation Trust, UK
Gertraud Morshead	Oxford Vaccine Group, Department of Paediatrics, University of Oxford, UK
Richard Morter	Jenner Institute, Nuffield Department of Medicine, University of Oxford, UK
Nathifa A. Moyo	Jenner Institute, Nuffield Department of Medicine, University of Oxford, UK
Mushiya Mpelembue	London Northwest University Healthcare, Northwick Park Hospital, London, UK
Ekta Mukhopadhyay	Jenner Institute, Nuffield Department of Medicine, University of Oxford, UK
Jilly Muller	Oxford Vaccine Group, Department of Paediatrics, University of Oxford, UK
Alasdair P.S. Munro	NIHR Southampton Clinical Research Facility, Southampton, UK
Sarah Murphy	Oxford Vaccine Group, Department of Paediatrics, University of Oxford, UK
Philomena Mweu	Oxford Vaccine Group, Department of Paediatrics, University of Oxford, UK
Gurudutt Naik	Aneurin Bevan University Health Board, Newport, Wales, UK
Kush Naker	NIHR/Wellcome Trust Birmingham Clinical Research Facility, Birmingham, UK
Eleni Nastouli	Clinical Microbiology and Virology Department, University College London Hospitals NHS Trust, UK
Cecilia Njenga	NIHR Imperial Clinical Research Facility, London, UK
Andrés Noé	Jenner Institute, Nuffield Department of Medicine, University of Oxford, UK
Fay L Nugent	Jenner Institute, Nuffield Department of Medicine, University of Oxford, UK
Katie O'Brien	Oxford Vaccine Group, Department of Paediatrics, University of Oxford, UK
Daniel O'Connor	Oxford Vaccine Group, Department of Paediatrics, University of Oxford, UK
Blanché Oguti	Oxford Vaccine Group, Department of Paediatrics, University of Oxford, UK
Victoria Olchawski	Clinical BioManufacturing Facility, Jenner Institute, University of Oxford, UK
Neil J Oldfield	School of Life Sciences, University of Nottingham, Nottingham, UK
Catarina Oliveira	Clinical BioManufacturing Facility, Jenner Institute, University of Oxford, UK
Peter J. O'Reilly	Oxford Vaccine Group, Department of Paediatrics, University of Oxford, UK
Piper Osborne	Oxford Vaccine Group, Department of Paediatrics, University of Oxford, UK
David R. J. Owen	NIHR Imperial Clinical Research Facility, London, UK
Daniel R. Owens	NIHR Southampton Clinical Research Facility, Southampton, UK
Nelly Owino	Oxford Vaccine Group, Department of Paediatrics, University of Oxford, UK
Mihaela Pacurar	NIHR Southampton Clinical Research Facility, Southampton, UK
Susan Palmer	Aneurin Bevan University Health Board, Newport, Wales, UK
Helena M. R. T. Parracho	Clinical BioManufacturing Facility, Jenner Institute, University of Oxford, UK
Dipak Patel	Clinical Research and Innovation Office, Sheffield Teaching Hospitals NHS Foundation Trust, UK
Maia Patrick-Smith	University of Oxford Medical School, Medical Sciences Division, University of Oxford, UK
Ruth O. Payne	Department of Infection and Tropical Medicine, Sheffield Teaching Hospitals NHS Foundation Trust and the Department of Infection, Immunity and Cardiovascular Disease, University of Sheffield, UK
Elizabeth J. Penn	National Infection Service, Public Health England, UK
Anna Pennington	Aneurin Bevan University Health Board, Newport, Wales, UK
Marco Polo Peralta Alvarez	Jenner Institute, Nuffield Department of Medicine, University of Oxford, UK
James Perring	University of Oxford Medical School, Medical Sciences Division, University of Oxford, UK

Angelina Peterson	Department of Clinical Sciences, Liverpool School of Tropical Medicine, UK
Jennifer Phillips	University Hospitals Bristol & Weston NHS Foundation Trust
Lorinda Pickup	NIHR Cambridge Clinical Research Facility, Cambridge, UK
Jo Piper	NIHR Cambridge Clinical Research Facility, Cambridge, UK
Dimitra Pipini	Jenner Institute, Nuffield Department of Medicine, University of Oxford, UK
Mary Plank	AstraZeneca BioPharmaceuticals PLC
Sinead Plant	Clinical Infection Research Group, NHS Lothian, Edinburgh, UK
Jennifer Pooley	North Bristol NHS Trust, Bristol, UK
Ian Poulton	Jenner Institute, Nuffield Department of Medicine, University of Oxford, UK
Claire Powers	Jenner Institute, Nuffield Department of Medicine, University of Oxford, UK
David A. Price	Department of Infection and Tropical Medicine, Newcastle upon Tyne Hospitals NHS Foundation Trust, UK
Vivien Price	NIHR/Wellcome Trust Birmingham Clinical Research Facility, Birmingham, UK
Pamela C. Proud	National Infection Service, Public Health England, UK
Samuel Provstgaard-Morys	Oxford Vaccine Group, Department of Paediatrics, University of Oxford, UK
David Pulido	Jenner Institute, Nuffield Department of Medicine, University of Oxford, UK
Sheena Quaid	London Northwest University Healthcare, Northwick Park Hospital, London, UK
Kajal Radia	University of Oxford Medical School, Medical Sciences Division, University of Oxford, UK
Durga Rajapaksa	National Infection Service, Public Health England, UK
Thurkka Rajeswaran	NIHR BRC at Guy's and St Thomas' NHS Foundation Trust, UK
Alberto San Francisco Ramos	Vaccine Institute, Institute of Infection & Immunity, St. Georges, University of London and St Georges University Hospitals NHS Trust, London, UK
Fernando Ramos Lopez	Jenner Institute, Nuffield Department of Medicine, University of Oxford, UK
Tommy Rampling	Clinical Microbiology and Virology Department, University College London Hospitals NHS Trust, UK
Isobel Ramsay	Infectious Diseases Department, Cambridge and Peterborough NHS Foundation Trust, UK
Jade Rand	NIHR Southampton Clinical Research Facility, Southampton, UK
Helen Ratcliffe	Oxford Vaccine Group, Department of Paediatrics, University of Oxford, UK
Pooja Ravji	Infectious Diseases Department, Cambridge and Peterborough NHS Foundation Trust, UK
Thomas Rawlinson	Jenner Institute, Nuffield Department of Medicine, University of Oxford, UK
David Rea	Clinical Research Network, West of England, UK
Ashwin Reddy	Pulmonary Vascular Diseases Unit, Cambridge and Peterborough NHS Foundation Trust, UK
Mila Resuello-Dauti	NIHR UCLH Clinical Research Facility, London, UK
Emilia Reyes Pabon	Clinical BioManufacturing Facility, Jenner Institute, University of Oxford, UK
Sarah Rhead	Oxford Vaccine Group, Department of Paediatrics, University of Oxford, UK
Tawassal Riaz	Infection Sciences, North Bristol NHS Trust, Bristol, UK
Carla M. Ribiero	NIHR Cambridge Clinical Research Facility, Cambridge, UK
Marivic Ricamara	NIHR UCLH Clinical Research Facility, London, UK
Alex Richter	NIHR/Wellcome Trust Birmingham Clinical Research Facility & Institute of Immunology and Immunotherapy, University of Birmingham, UK
Neil D. Ritchie	Department of Infectious Diseases, Queen Elizabeth University Hospital, Glasgow, UK

Adam J. Ritchie	Jenner Institute, Nuffield Department of Medicine, University of Oxford, UK
Alexander J. Robbins	NIHR Imperial Clinical Research Facility, London, UK
Hannah Roberts	Oxford Vaccine Group, Department of Paediatrics, University of Oxford, UK
Ryan E Robinson	Department of Clinical Sciences, Liverpool School of Tropical Medicine and Liverpool University Hospitals NHS Foundation Trust, UK
Sophie Roche	University of Oxford Medical School, Medical Sciences Division, University of Oxford, UK
Christine S. Rollier	Oxford Vaccine Group, Department of Paediatrics, University of Oxford, UK
Louisa Rose	Jenner Institute, Nuffield Department of Medicine, University of Oxford, UK
Amy L. Ross Russell	NIHR Southampton Clinical Research Facility, Southampton, UK
Simon Royal	School of Medicine, Division of Primary Care, University of Nottingham, Nottingham, UK
Indra Rudiansyah	Jenner Institute, Nuffield Department of Medicine, University of Oxford, UK
Kim Ryalls	Pharmacy Department, Sheffield Teaching Hospitals NHS Foundation Trust
Charlotte E. Sabine	NIHR/Wellcome Trust Birmingham Clinical Research Facility, Birmingham, UK
Stephen Saich	NIHR Southampton Clinical Research Facility, Southampton, UK
Jessica C Sale	NIHR/Wellcome Trust Birmingham Clinical Research Facility, Birmingham, UK
Ahmed M. Salman	Jenner Institute, Nuffield Department of Medicine, University of Oxford, UK
Stephannie Salvador	Jenner Institute, Nuffield Department of Medicine, University of Oxford, UK
Amada Sanchez-Gonzalez	Department of Infection and Tropical Medicine, Newcastle upon Tyne Hospitals NHS Foundation Trust, UK
Helen Sanders	Jenner Institute, Nuffield Department of Medicine, University of Oxford, UK
Katherine Sanders	Oxford Vaccine Group, Department of Paediatrics, University of Oxford, UK
Iman Satti	Jenner Institute, Nuffield Department of Medicine, University of Oxford, UK
Jack E. Saunders	Jenner Institute, Nuffield Department of Medicine, University of Oxford, UK
Caroline Saunders	NIHR Cambridge Clinical Research Facility, Cambridge, UK
Ina Schim van der Loeff	Department of Infection and Tropical Medicine, Newcastle upon Tyne Hospitals NHS Foundation Trust and Translational and Clinical Research Institute, Immunity and Inflammation Theme, Newcastle University
Ella Schofield	University of Oxford Medical School, Medical Sciences Division, University of Oxford, UK
Gavin R. Screaton	Medical Sciences, University of Oxford, UK
Samiullah Seddiqi	Oxford Vaccine Group, Department of Paediatrics, University of Oxford, UK
Rameswara R. Segireddy	Jenner Institute, Nuffield Department of Medicine, University of Oxford, UK
Sonia Serrano	NIHR BRC at Guy's and St Thomas' NHS Foundation Trust, UK
Sifut Sethi	Hull University Teaching Hospitals NHS Trust, Hull, UK
Farah Shahi	Hull University Teaching Hospitals NHS Trust, Hull, UK
Imam Shaik	National Infection Service, Public Health England, UK
Hannah R. Sharpe	Jenner Institute, Nuffield Department of Medicine, University of Oxford, UK
Katherine Sharrocks	Department of Medicine, University of Cambridge, UK
Robert Shaw	Oxford Vaccine Group, Department of Paediatrics, University of Oxford, UK
Emma Sheehan	Jenner Institute, Nuffield Department of Medicine, University of Oxford, UK
Amy Shepherd	Clinical Infection Research Group, Regional Infectious Diseases Unit, NHS Lothian, UK
Farah Shiham	Department of Clinical Sciences, Liverpool School of Tropical Medicine and Liverpool University Hospitals NHS Foundation Trust, UK
Sarah E. Silk	Jenner Institute, Nuffield Department of Medicine, University of Oxford, UK

Laura Silva-Reyes	Oxford Vaccine Group, Department of Paediatrics, University of Oxford, UK
Nisha Singh	Oxford Vaccine Group, Department of Paediatrics, University of Oxford, UK
Jaisi Sinha	Public Health Wales NHS Trust, Cardiff, UK
Holly E. Smith	Jenner Institute, Nuffield Department of Medicine, University of Oxford, UK
David J Smith	Oxford Vaccine Group, Department of Paediatrics, University of Oxford, UK
Catherine C Smith	Oxford Vaccine Group, Department of Paediatrics, University of Oxford, UK
Carla Solórzano	Department of Clinical Sciences, Liverpool School of Tropical Medicine, UK
Kim Sorley	NIHR Imperial Clinical Research Facility, London, UK
Luciana Sowole	NIHR BRC at Guy's and St Thomas' NHS Foundation Trust, UK
Alexandra J Spencer	Jenner Institute, Nuffield Department of Medicine, University of Oxford, UK
Lisa Stockdale	Oxford Vaccine Group, Department of Paediatrics, University of Oxford, UK
Lisa V. Stockwell	Oxford Vaccine Group, Department of Paediatrics, University of Oxford, UK
Arabella S. V. Stuart	Oxford Vaccine Group, Department of Paediatrics, University of Oxford, UK
Ann Sturdy	London Northwest University Healthcare, Northwick Park Hospital, London, UK
Joe Suich	Hull University Teaching Hospitals NHS Trust, Hull, UK
Natalina Sutton	Vaccine Institute, Institute of Infection & Immunity, St. Georges, University of London and St Georges University Hospitals NHS Trust, London, UK
Anna Szigeti	Oxford Vaccine Group, Department of Paediatrics, University of Oxford, UK
Abdessamad Tahiri-Alaoui	Clinical BioManufacturing Facility, Jenner Institute, University of Oxford, UK
Farah Tahmasebi	Manchester Lighthouse Laboratory, Manchester, UK
Rachel Tanner	Jenner Institute, Nuffield Department of Medicine, University of Oxford, UK
Alexander W Tarr	School of Life Sciences, University of Nottingham, Nottingham, UK & NIHR Nottingham Biomedical Research Centre, Nottingham University Hospitals NHS Trust, Nottingham, UK
Richard Tarrant	Clinical BioManufacturing Facility, Jenner Institute, University of Oxford, UK
Natalie Tate	Department of Clinical Sciences, Liverpool School of Tropical Medicine, UK
Keja Taylor	Clinical BioManufacturing Facility, Jenner Institute, University of Oxford, UK
Iona Jennifer Taylor	Jenner Institute, Nuffield Department of Medicine, University of Oxford, UK
Justin Taylor	Oxford Vaccine Group, Department of Paediatrics, University of Oxford, UK
Rebecca te Water Naude	University of Oxford Medical School, Medical Sciences Division, University of Oxford, UK
Kate Templeton	Clinical Infection Research Group, NHS Lothian, Edinburgh, UK
Yrene Themistocleous	Jenner Institute, Nuffield Department of Medicine, University of Oxford, UK
Merin Thomas	Jenner Institute, Nuffield Department of Medicine, University of Oxford, UK
Kelly M Thomas	National Infection Service, Public Health England, UK
Tonia M Thomas	Oxford Vaccine Group, Department of Paediatrics, University of Oxford, UK
Julia Thompson	AstraZeneca BioPharmaceuticals PLC
Amber J Thompson	Oxford Vaccine Group, Department of Paediatrics, University of Oxford, UK
Patrick J Tighe	School of Life Sciences, University of Nottingham, Nottingham, UK
Gerlynn Ferreras Tiongson	London Northwest University Healthcare, Northwick Park Hospital, London, UK
Adriana Tomic	Oxford Vaccine Group, Department of Paediatrics, University of Oxford, UK
Estee Torok	Departments of Infectious Diseases and Microbiology, Cambridge University Hospitals NHS Foundation Trust; Cambridge, UK

James Towner	University of Oxford Medical School, Medical Sciences Division, University of Oxford, UK
Nguyen Tran	Jenner Institute, Nuffield Department of Medicine, University of Oxford, UK
Julia A. Tree	National Infection Service, Public Health England, UK
Gerardo Trillana	NIHR BRC at Guy's and St Thomas' NHS Foundation Trust, UK
Charlotte Trinham	NIHR/Wellcome Trust Birmingham Clinical Research Facility, Birmingham, UK
Rose Trivett	Oxford Vaccine Group, Department of Paediatrics, University of Oxford, UK
Adam Truby	Jenner Institute, Nuffield Department of Medicine, University of Oxford, UK
Aadil El-Turabi	Jenner Institute, Nuffield Department of Medicine, University of Oxford, UK
Richard Turner	AstraZeneca BioPharmaceuticals PLC
Cheryl Turner	Jenner Institute, Nuffield Department of Medicine, University of Oxford, UK
Nicola Turner	NIHR UCLH Clinical Research Facility, London, UK
Bhavya Tyagi	London Northwest University Healthcare, Northwick Park Hospital, London, UK
Marta Ulaszewska	Jenner Institute, Nuffield Department of Medicine, University of Oxford, UK
Benjamin R. Underwood	Windsor Research Unit, Cambridge and Peterborough NHS Foundation Trust, UK
Maithili Varadarajan	Hull University Teaching Hospitals NHS Trust, Hull, UK
Marije K Verheul	Oxford Vaccine Group, Department of Paediatrics, University of Oxford, UK
Iason Vichos	Oxford Vaccine Group, Department of Paediatrics, University of Oxford, UK
Laura Walker	Oxford Vaccine Group, Department of Paediatrics, University of Oxford, UK
Matthew E Wand	National Infection Service, Public Health England, UK
Sarah C. Warren	NIHR Southampton Clinical Research Facility, Southampton, UK
Marion E. E. Watson	Jenner Institute, Nuffield Department of Medicine, University of Oxford, UK
Ekaterina Watson	London Northwest University Healthcare, Northwick Park Hospital, London, UK
Stewart Webb	Department of Infectious Diseases, Queen Elizabeth University Hospital, Glasgow, UK
Andrea Webster	Research Directorate, Newcastle upon Tyne Hospitals NHS Foundation Trust, UK
Rowena Weighell	NIHR Cambridge Clinical Research Facility, Cambridge, UK
Jeanette H. Wells	Aneurin Bevan University Health Board, Newport, Wales, UK
Beth White	Department of Infectious Diseases, Queen Elizabeth University Hospital, Glasgow, UK
Rachel White	Oxford Vaccine Group, Department of Paediatrics, University of Oxford, UK
Caroline White	Oxford Vaccine Group, Department of Paediatrics, University of Oxford, UK
Paul Williams	Clinical BioManufacturing Facility, Jenner Institute, University of Oxford, UK
Rachel L Williams	Research & Innovation, North Bristol NHS Trust, Bristol, UK
Rebecca L. Winslow	NIHR/Wellcome Trust Birmingham Clinical Research Facility, Birmingham, UK
Danielle Woods	Jenner Institute, Nuffield Department of Medicine, University of Oxford, UK
Andrew T. Worth	Jenner Institute, Nuffield Department of Medicine, University of Oxford, UK
Daniel Wright	Jenner Institute, Nuffield Department of Medicine, University of Oxford, UK
Marzena Wroblewska	Jenner Institute, Nuffield Department of Medicine, University of Oxford, UK
Xin Li Yao	Oxford Vaccine Group, Department of Paediatrics, University of Oxford, UK
Yee Ting Nicole Yim	NIHR UCLH Clinical Research Facility, London, UK
Dalila Zizi	Clinical BioManufacturing Facility, Jenner Institute, University of Oxford, UK

The COVID-19 Genomics UK (COG-UK) Consortium

Funding acquisition, Leadership and supervision, Metadata curation, Project administration, Samples and logistics, Sequencing and analysis, Software and analysis tools, and Visualisation:
Dr Samuel C Robson PhD [13].

Funding acquisition, Leadership and supervision, Metadata curation, Project administration, Samples and logistics, Sequencing and analysis, and Software and analysis tools:
Prof Nicholas J Loman PhD [41] and Dr Thomas R Connor PhD [10, 69].

Leadership and supervision, Metadata curation, Project administration, Samples and logistics, Sequencing and analysis, Software and analysis tools, and Visualisation:
Dr Tanya Golubchik PhD [5].

Funding acquisition, Metadata curation, Samples and logistics, Sequencing and analysis, Software and analysis tools, and Visualisation:
Dr Rocio T Martinez Nunez PhD [42].

Funding acquisition, Leadership and supervision, Metadata curation, Project administration, and Samples and logistics:
Dr Catherine Ludden PhD [88].

Funding acquisition, Leadership and supervision, Metadata curation, Samples and logistics, and Sequencing and analysis:
Dr Sally Corden PhD [69].

Funding acquisition, Leadership and supervision, Project administration, Samples and logistics, and Sequencing and analysis:
Ian Johnston [99] and Dr David Bonsall PhD [5].

Funding acquisition, Leadership and supervision, Sequencing and analysis, Software and analysis tools, and Visualisation:
Prof Colin P Smith PhD [87] and Dr Ali R Awan PhD [28].

Funding acquisition, Samples and logistics, Sequencing and analysis, Software and analysis tools, and Visualisation:
Dr Giselda Bucca PhD [87].

Leadership and supervision, Metadata curation, Project administration, Samples and logistics, and Sequencing and analysis:
Dr M. Estee Torok FRCP [22, 101].

Leadership and supervision, Metadata curation, Project administration, Samples and logistics, and Visualisation:
Dr Kordo Saeed MD/ FRCPath [81, 110] and Dr Jacqui A Prieto PhD [83, 109].

Leadership and supervision, Metadata curation, Project administration, Sequencing and analysis, and Software and analysis tools:
Dr David K Jackson PhD [99].

Metadata curation, Project administration, Samples and logistics, Sequencing and analysis, and Software and analysis tools:
Dr William L Hamilton PhD [22].
Metadata curation, Project administration, Samples and logistics, Sequencing and analysis, and Visualisation:
Dr Luke B Snell MSc/ MBBS [11].

Funding acquisition, Leadership and supervision, Metadata curation, and Samples and logistics:
Dr Catherine Moore [69].

Funding acquisition, Leadership and supervision, Project administration, and Samples and logistics:
Dr Ewan M Harrison PhD [99, 88].

Leadership and supervision, Metadata curation, Project administration, and Samples and logistics:
Dr Sonia Goncalves PhD [99].

Leadership and supervision, Metadata curation, Samples and logistics, and Sequencing and analysis:
Prof Ian G Goodfellow PhD [24], Dr Derek J Fairley PhD [3, 72], Prof Matthew W Loose PhD [18] and Joanne Watkins MSc [69].

Leadership and supervision, Metadata curation, Samples and logistics, and Software and analysis tools:
Rich Livett MSc [99].

Leadership and supervision, Metadata curation, Samples and logistics, and Visualisation:
Dr Samuel Moses MD [25, 106].

Leadership and supervision, Metadata curation, Sequencing and analysis, and Software and analysis tools:
Dr Roberto Amato PhD [99], Dr Sam Nicholls PhD [41] and Dr Matthew Bull PhD [69].

Leadership and supervision, Project administration, Samples and logistics, and Sequencing and analysis:
Prof Darren L Smith PhD [37, 58, 105].

Leadership and supervision, Sequencing and analysis, Software and analysis tools, and Visualisation:
Dr Jeff Barrett PhD [99] and Prof David M Aanensen PhD [14, 114].

Metadata curation, Project administration, Samples and logistics, and Sequencing and analysis:
Dr Martin D Curran PhD [65], Dr Surendra Parmar PhD [65], Dr Dinesh Aggarwal MRCP [95, 99, 64] and Dr James G Shepherd MBChB/MRCP [48].

Metadata curation, Project administration, Sequencing and analysis, and Software and analysis tools:
Dr Matthew D Parker PhD [93].

Metadata curation, Samples and logistics, Sequencing and analysis, and Visualisation:
Dr Sharon Glaysher PhD [61].

Metadata curation, Sequencing and analysis, Software and analysis tools, and Visualisation:
Dr Matthew Bashton PhD [37, 58], Dr Anthony P Underwood PhD [14, 114], Dr Nicole Pacchiarini PhD [69] and Dr Katie F Loveson PhD [77].

Project administration, Sequencing and analysis, Software and analysis tools, and Visualisation:

Dr Alessandro M Carabelli PhD [88].

Funding acquisition, Leadership and supervision, and Metadata curation:
Dr Kate E Templeton PhD [53, 90].

Funding acquisition, Leadership and supervision, and Project administration:
Dr Cordelia F Langford PhD [99], John Sillitoe BEng [99], Dr Thushan I de Silva PhD [93] and Dr Dennis Wang PhD [93].

Funding acquisition, Leadership and supervision, and Sequencing and analysis:
Prof Dominic Kwiatkowski [99, 107], Prof Andrew Rambaut DPhil [90], Dr Justin O'Grady PhD [70, 89] and Dr Simon Cottrell PhD [69].

Leadership and supervision, Metadata curation, and Sequencing and analysis:
Prof Matthew T.G. Holden PhD [68] and Prof Emma C Thomson PhD/FRCP [48].

Leadership and supervision, Project administration, and Samples and logistics:
Dr Husam Osman PhD [64, 36], Dr Monique Andersson PhD [59], Prof Anoop J Chauhan [61] and Dr Mohammed O Hassan-Ibrahim PhD/FRCPath [6].

Leadership and supervision, Project administration, and Sequencing and analysis:
Dr Mara Lawniczak [99].

Leadership and supervision, Samples and logistics, and Sequencing and analysis:
Prof Ravi Kumar Gupta PhD [88, 113], Dr Alex Alderton PhD [99], Dr Meera Chand [66], Dr Chrystala Constantinidou PhD [94], Dr Meera Unnikrishnan PhD [94], Prof Alistair C Darby PhD [92], Prof Julian A Hiscox PhD [92] and Prof Steve Paterson PhD [92].

Leadership and supervision, Sequencing and analysis, and Software and analysis tools:
Dr Inigo Martincorena [99], Prof David L Robertson PhD [48], Dr Erik M Volz PhD [39], Dr Andrew J Page PhD [7] and Prof Oliver G Pybus DPhil [23].

Leadership and supervision, Sequencing and analysis, and Visualisation:
Dr Andrew R Bassett PhD [99].

Metadata curation, Project administration, and Samples and logistics:
Dr Cristina V Ariani PhD [99], Dr Michael H Spencer Chapman MBBS [99, 88], Dr Kathy K Li MBBCh/FRCPath [48], Dr Rajiv N Shah BMBS/MRCP/MSc [48], Dr Natasha G Jesudason MBChB MRCP FRCPath [48] and Dr Yusri Taha MD/PhD [50].

Metadata curation, Project administration, and Sequencing and analysis:
Martin P McHugh MSc [53], Dr Rebecca Dewar PhD [53].

Metadata curation, Samples and logistics, and Sequencing and analysis:
Dr Aminu S Jahun PhD [24], Dr Claire McMurray PhD [41], Ms Sarojini Pandey MSc [84], Dr James P McKenna PhD [3], Dr Andrew Nelson PhD [58, 105], Dr Gregory R Young PhD [37, 58], Dr Clare M McCann PhD [58, 105] and Mr Scott Elliott [61].

Metadata curation, Samples and logistics, and Visualisation:
Ms Hannah Lowe MSc [25].

Metadata curation, Sequencing and analysis, and Software and analysis tools:
Dr Ben Temperton Ph.D. [91], Dr Sunando Roy PhD [82], Dr Anna Price PhD [10], Dr Sara Rey PhD [69] and Mr Matthew Wyles [93].

Metadata curation, Sequencing and analysis, and Visualisation:
Stefan Rooke MSc [90] and Dr Sharif Shaaban PhD [68].

Project administration, Samples and logistics, Sequencing and analysis:
Dr Mariateresa de Cesare PhD [98].

Project administration, Samples and logistics, and Software and analysis tools:
Laura Letchford BSc [99].

Project administration, Samples and logistics, and Visualisation:
Miss Siona Silveira MSc [81], Dr Emanuela Pelosi FRCPath [81] and Dr Eleri Wilson-Davies MD/FRCPath [81].

Samples and logistics, Sequencing and analysis, and Software and analysis tools:
Dr Myra Hosmillo PhD [24].

Sequencing and analysis, Software and analysis tools, and Visualisation:
Áine O'Toole MSc [90], Dr Andrew R Hesketh PhD [87], Mr Richard Stark MSc [94], Dr Louis du Plessis PhD [23], Dr Chris Ruis PhD [88], Dr Helen Adams PhD [4] and Dr Yann Bourgeois PhD [76].

Funding acquisition, and Leadership and supervision:
Dr Stephen L Michell PhD [91], Prof Dimitris Grammatopoulos PhD/FRCPath [84, 112], Dr Jonathan Edgeworth PhD/FRCPath [12], Prof Judith Breuer MD [30, 82], Prof John A Todd PhD [98] and Dr Christophe Fraser PhD [5].

Funding acquisition, and Project administration:
Dr David Buck PhD [98] and Michaela John BSc [9].

Leadership and supervision, and Metadata curation:
Dr Gemma L Kay PhD [70].

Leadership and supervision, and Project administration:
Steve Palmer [99], Prof Sharon J Peacock [88, 64] and David Heyburn [69].

Leadership and supervision, and Samples and logistics:
Danni Weldon BSc [99], Dr Esther Robinson PhD [64, 36], Prof Alan McNally PhD [41, 86], Dr Peter Muir PhD [64], Dr Ian B Vipond PhD [64], Dr John BoYes MBChB [29], Dr Venkat Sivaprakasam PhD [46], Dr Tranprit Saluja FRCPath/MD [75], Dr Samir Dervisevic FRCPath [54] and Dr Emma J Meader FRCPath [54].

Leadership and supervision, and Sequencing and analysis:
Dr Naomi R Park PhD [99], Karen Oliver BSc [99], Dr Aaron R Jeffries Ph.D. [91], Dr Sascha Ott PhD [94], Dr Ana da Silva Filipe PhD [48], Dr David A Simpson PhD [72] and Dr Chris Williams MB BS [69].

Leadership and supervision, and Visualisation:
Dr Jane AH Masoli MBChB [73, 91].

Metadata curation, and Samples and logistics:
Dr Bridget A Knight PhD. [73, 91], Dr Christopher R Jones Ph.D. [73, 91], Mr Cherian Koshy MSc CSci FIBMS [1], Miss Amy Ash BSc [1], Dr Anna Casey PhD [71], Dr Andrew Bosworth PhD [64, 36], Dr Liz Ratcliffe PhD [71], Dr Li Xu-McCrae PhD [36], Miss Hannah M Pymont MSc [64], Ms Stephanie Hutchings [64], Dr Lisa Berry PhD [84], Ms Katie Jones MSc [84], Dr Fenella Halstead PhD [46], Mr Thomas Davis MSc [21], Dr Christopher Holmes PhD [16], Prof Miren Iturriza-Gomara PhD [92], Dr Anita O Lucaci PhD [92], Dr Paul Anthony Randell MBBCh [38, 104], Dr Alison Cox PhD [38, 104], Pinglawathee Madona [38, 104], Dr Kathryn Ann Harris PhD [30], Dr Julianne Rose Brown PhD [30], Dr Tabitha W Mahungu FRCPath [74], Dr

Dianne Irish-Tavares FRCPath [74], Dr Tanzina Haque FRCPath PhD [74], Dr Jennifer Hart MRCP [74], Mr Eric Witele MSc [74], Mrs Melisa Louise Fenton DipHE [75], Mr Steven Liggett [79], Dr Clive Graham MD [56], Ms Emma Swindells Bsc [57], Ms Jennifer Collins BSc [50], Mr Gary Eltringham BSc [50], Ms Sharon Campbell MSc [17], Dr Patrick C McClure PhD [97], Dr Gemma Clark PhD [15], Dr Tim J Sloan PhD [60], Mr Carl Jones [15] and Dr Jessica Lynch PhD MBChB [2, 111].

Metadata curation, and Sequencing and analysis:
Dr Ben Warne MRCP [8], Steven Leonard PhD [99], Jillian Durham BSc [99], Dr Thomas Williams MD [90], Dr Sam T Haldenby PhD [92], Dr Nathaniel Storey PhD [30], Dr Nabil-Fareed Alikhan PhD [70], Dr Nadine Holmes PhD [18], Dr Christopher Moore PhD [18], Mr Matthew Carlile BSc [18], Malorie Perry MSc [69], Dr Noel Craine DPhil [69], Prof Ronan A Lyons MD [80], Miss Angela H Beckett MSc [13], Salman Goudarzi PhD [77], Christopher Fearn MRes [77], Kate Cook [77], Hannah Dent BSc [77] and Hannah Paul MRes [77].

Metadata curation, and Software and analysis tools:
Robert Davies [99].

Project administration, and Samples and logistics:
Beth Blane BSc [88], Sophia T Girgis MSc [88], Dr Mathew A Beale PhD [99], Katherine L Bellis [99, 88], Matthew J Dorman [99], Eleanor Drury [99], Leanne Kane [99], Sally Kay [99], Dr Samantha McGuigan [99], Dr Rachel Nelson PhD [99], Liam Prestwood [99], Dr Shavanthi Rajatileka PhD [99], Dr Rahul Batra MD [12], Dr Rachel J Williams PhD [82], Dr Mark Kristiansen PhD [82], Dr Angie Green PhD [98], Miss Anita Justice MSc [59], Dr Adhyana I.K Mahanama MD [81, 102] and Dr Buddhini Samaraweera MD [81, 102].

Project administration, and Sequencing and analysis:
Dr Nazreen F Hadjirin PhD [88] and Dr Joshua Quick PhD [41].

Project administration, and Software and analysis tools:
Mr Radoslaw Poplawski BSc [41].

Samples and logistics, and Sequencing and analysis:
Leanne M Kermack MSc [88], Nicola Reynolds PhD [7], Grant Hall BS [24], Yasmin Chaudhry BSc [24], Malte L Pinckert MPhil [24], Dr Iliana Georgana PhD [24], Dr Robin J Moll PhD [99], Dr Alicia Thornton [66], Dr Richard Myers [66], Dr Joanne Stockton PhD [41], Miss Charlotte A Williams BSc [82], Dr Wen C Yew PhD [58], Alexander J Trotter MRes [70], Miss Amy Trebes MSc [98], Mr George MacIntyre-Cockett BSc [98], Alec Birchley MSc [69], Alexander Adams BSc [69], Amy Plimmer [69], Bree Gatica-Wilcox MPhil [69], Dr Caoimhe McKerr PhD [69], Ember Hilvers MA [69], Hannah Jones [69], Dr Hibo Asad PhD [69], Jason Coombes BSc [69], Johnathan M Evans MSc [69], Laia Fina [69], Lauren Gilbert A-Levels [69], Lee Graham BSc [69], Michelle Cronin [69], Sara Kumziene-SummerhaYes MSc [69], Sarah Taylor [69], Sophie Jones MSc [69], Miss Danielle C Groves BA [93], Mrs Peijun Zhang MSc [93], Miss Marta Gallis MSc [93] and Miss Stavroula F Louka MSc [93].

Samples and logistics, and Software and analysis tools:
Dr Igor Starinskij Msc MRCP [48].

Sequencing and analysis, and Software and analysis tools:
Dr Chris J Illingworth PhD [47], Dr Chris Jackson PhD [47], Ms Marina Gourtovaia MSc [99], Gerry Tonkin-Hill [99], Kevin Lewis [99], Dr Jaime M Tovar-Corona PhD [99], Dr Keith James PhD [99], Dr Laura Baxter PhD [94], Dr Mohammad T. Alam PhD [94], Dr Richard J Orton PhD [48], Dr Joseph Hughes PhD [48], Dr Sreenu Vattipally PhD [48], Dr Manon Ragonnet-Cronin PhD [39], Dr Fabricia F. Nascimento PhD [39], Mr David Jorgensen MSc [39], Ms Olivia Boyd MSc [39], Ms Lily Geidelberg MSc [39], Dr Alex E Zarebski PhD [23], Dr Jayna Raghwani PhD [23], Dr Moritz UG Kraemer DPhil [23], Joel Southgate MSc [10, 69], Dr Benjamin B Lindsey MRCP [93] and Mr Timothy M Freeman MPhil [93].

Software and analysis tools, and Visualisation:

Jon-Paul Keatley [99], Dr Joshua B Singer PhD [48], Leonardo de Oliveira Martins PhD [70], Dr Corin A Yeats PhD [14], Dr Khalil Abudahab PhD [14, 114], Mr Ben EW Taylor MEng [14, 114] and Mirko Menegazzo [14].

Leadership and supervision:
Prof John Danesh [99], Wendy Hogsden MSc [46], Dr Sahar Eldirdiri MBBS MSc FRCPath [21], Mrs Anita Kenyon MSc [21], Dr Jenifer Mason MBBS [43], Mr Trevor I Robinson MSc [43], Prof Alison Holmes MD [38, 103], Dr James Price PhD [38, 103], Prof John A Hartley PhD [82], Dr Tanya Curran PhD [3], Dr Alison E Mather PhD [70], Dr Giri Shankar [69], Dr Rachel Jones [69], Dr Robin Howe [69] and Dr Sian Morgan FRCPath [9].

Metadata curation:
Dr Elizabeth Wastenge MD [53], Dr Michael R Chapman PhD [34, 88, 99], Mr Siddharth Mookerjee MPH [38, 103], Dr Rachael Stanley PhD [54], Mrs Wendy Smith [15], Prof Timothy Peto PhD [59], Dr David Eyre PhD [59], Dr Derrick Crook [59], Dr Gabrielle Vernet MBBS [33], Dr Christine Kitchen PhD [10], Huw Gulliver [10], Dr Ian Merrick PhD [10], Prof Martyn Guest PhD [10], Robert Munn BSc [10], Dr Declan T Bradley [63, 72] and Dr Tim Wyatt [63].

Project administration:
Dr Charlotte Beaver [99], Luke Foulser [99], Sophie Palmer [88], Carol M Churcher [88], Ellena Brooks MA [88], Kim S Smith [88], Dr Katerina Galai PhD [88], Georgina M McManus BSc [88], Dr Frances Bolt PhD [38, 103], Dr Francesc Coll PhD [19], Lizzie Meadows MA [70], Dr Stephen W Attwood PhD [23], Dr Alisha Davies [69], Elen De Lacy MSc [69], Fatima Downing [69], Sue Edwards [69], Dr Garry P Scarlett PhD [76], Mrs Sarah Jeremiah MSc [83] and Dr Nikki Smith PhD [93].

Samples and logistics:
Danielle Leek Bsc [88], Sushmita Sridhar BS [88, 99], Sally Forrest BSc [88], Claire Cormie [88], Harmeet K Gill PhD [88], Joana Dias MSc [88], Ellen E Higginson PhD [88], Mailis Maes MPhil [88], Jamie Young BSc [88], Michelle Wantoch PhD [7], Sanger Covid Team (www.sanger.ac.uk/covid-team) [99], Dorota Jamrozy [99], Stephanie Lo [99], Dr Minal Patel PhD [99], Verity Hill [90], Ms Claire M Bewshea MSc [91], Prof Sian Ellard FRCPath [73, 91], Dr Cressida Auckland FRCPath [73], Dr Ian Harrison [66], Dr Chloe Bishop [66], Dr Vicki Chalker [66], Dr Alex Richter PhD [85], Dr Andrew Beggs PhD [85], Dr Angus Best PhD [86], Dr Benita Percival PhD [86], Dr Jeremy Mirza PhD [86], Dr Oliver Megram PhD [86], Dr Megan Mayhew PhD [86], Dr Liam Crawford PhD [86], Dr Fiona Ashcroft PhD [86], Dr Emma Moles-Garcia PhD [86], Dr Nicola Cumley PhD [86], Mr Richard Hopes [64], Dr Patawee Asamaphan PhD [48], Mr Marc O Niebel MSc [48], Prof Rory N Gunson PhD FRCPath [100], Dr Amanda Bradley PhD [52], Dr Alasdair Maclean PhD [52], Dr Guy Mollett MBChB [52], Dr Rachel Blacow MBChB [52], Mr Paul Bird MSc [16], Mr Thomas Helmer [16], Miss Karlie Fallon [16], Dr Julian Tang [16], Dr Antony D Hale MBBS [49], Dr Louissa R Macfarlane-Smith PhD [49], Katherine L Harper MBiol [49], Miss Holli Carden MSc [49], Dr Nicholas W Machin MSc [45, 64], Ms Kathryn A Jackson MSc [92], Dr Shazaad S Y Ahmad MSc [45, 64], Dr Ryan P George PhD [45], Dr Lance Turtle PhD MRCP [92], Mrs Elaine O'Toole BSc [43], Mrs Joanne Watts BSc [43], Mrs Cassie Breen BSc [43], Mrs Angela Cowell MSc [43], Ms Adela Alcolea-Medina [32, 96], Ms Themoula Charalampous MSc [12, 42], Amita Patel [11], Dr Lisa J Levett PhD [35], Dr Judith Heaney PhD [35], Dr Aileen Rowan PhD [39], Prof Graham P Taylor DSc [39], Dr Divya Shah PhD [30], Miss Laura Atkinson MSc [30], Mr Jack CD Lee MSc [30], Mr Adam P Westhorpe BSc [82], Dr Riaz Jannoo PhD [82], Dr Helen L Lowe PhD [82], Miss Angeliki Karamani MSc [82], Miss Leah Ensell BSc [82], Mrs Wendy Chatterton MSc [35], Miss Monika Pusok MSc [35], Mrs Ashok Dadrah MSc [75], Miss Amanda Symmonds MSc [75], Dr Graciela Sluga MD/MSC [44], Dr Zoltan Molnar PhD [72], Mr Paul Baker MD [79], Prof Stephen Bonner [79], Ms Sarah Essex [79], Dr Edward Barton MD [56], Ms Debra Padgett BSc [56], Ms Garren Scott BSc [56], Ms Jane Greenaway MSc [57], Dr Brendan AI Payne MD [50], Dr Shirelle Burton-Fanning MD [50], Dr Sheila Waugh MD [50], Dr Veena Raviprakash MD [17], Ms Nicola Sheriff BSc [17], Ms Victoria Blakey BSc [17], ms Lesley-Anne Williams BSc [17], Dr Jonathan Moore MD [27], Ms Susanne Stonehouse BSc [27], Dr Louise Smith [55], Dr Rose K Davidson PhD [89], Dr Luke Bedford [26], Dr Lindsay Coupland PhD [54], Ms Victoria Wright BSc [18], Dr Joseph G Chappell PhD [97], Dr Theocharis Tsoleridis PhD [97], Prof Jonathan Ball PhD [97], Mrs Manjinder Khakh [15], Dr Vicki M Fleming PhD [15], Dr Michelle M Lister PhD [15], Dr Hannah C

Howson-Wells PhD [15], Dr Louise Berry [15], Dr Tim Boswell [15], Dr Amelia Joseph [15], Dr Iona Willingham [15], Dr Nichola Duckworth [60], Dr Sarah Walsh [60], Dr Emma Wise PhD [2, 111], Dr Nathan Moore PhD [2, 111], Miss Matilde Mori BSc [2, 108, 111], Dr Nick Cortes MRCP FRCPath [2, 111], Dr Stephen Kidd PhD [2, 111], Dr Rebecca Williams BMBS [33], Laura Gifford MSc [69], Miss Kelly Bicknell [61], Dr Sarah Wyllie [61], Miss Allyson Lloyd [61], Mr Robert Impey MSc [61], Ms Cassandra S Malone MSc [6], Mr Benjamin J Cogger BSc [6], Nick Levene MSc [62], Lynn Monaghan [62], Dr Alexander J Keeley MRCP [93], Dr David G Partridge FRCP FRCPath [78, 93], Dr Mohammad Raza [78, 93], Dr Cariad Evans [78, 93] and Dr Kate Johnson [78, 93].

Sequencing and analysis:
Emma Betteridge BSc [99], Ben W Farr BSc [99], Scott Goodwin MSc [99], Dr Michael A Quail PhD [99], Carol Scott [99], Lesley Shirley MSc [99], Scott AJ Thurston BSc [99], Diana Rajan MSc [99], Dr Iraad F Bronner PhD [99], Louise Aigrain PhD [99], Dr Nicholas M Redshaw PhD [99], Dr Stefanie V Lensing PhD [99], Shane McCarthy [99], Alex Makunin [99], Dr Carlos E Balcazar PhD [90], Dr Michael D Gallagher PhD [90], Dr Kathleen A Williamson PhD [90], Thomas D Stanton BSc [90], Ms Michelle L Michelsen BSc [91], Ms Joanna Warwick-Dugdale BSc [91], Dr Robin Manley Ph.D. [91], Ms Audrey Farbos MSc [91], Dr James W Harrison Ph.D. [91], Dr Christine M Sambles Ph.D. [91], Dr David J Studholme PhD. [91], Dr Angie Lackenby [66], Dr Tamyo Mbisa [66], Dr Steven Platt [66], Mr Shahjahan Miah [66], Dr David Bibby [66], Dr Carmen Manso [66], Dr Jonathan Hubb [66], Dr Gavin Dabrera [66], Dr Mary Ramsay [66], Dr Daniel Bradshaw [66], Dr Ulf Schaefer [66], Dr Natalie Groves [66], Dr Eileen Gallagher [66], Dr David Lee [66], Dr David Williams [66], Dr Nicholas Ellaby [66], Hassan Hartman [66], Nikos Manesis [66], Vineet Patel [66], Juan Ledesma [67], Ms Katherine A Twohig [67], Dr Elias Allara [64, 88], Ms Clare Pearson [64, 88], Mr Jeffrey K. J. Cheng MSc [94], Dr Hannah E. Bridgewater PhD [94], Ms Lucy R. Frost BSc [94], Ms Grace Taylor-Joyce BSc [94], Dr Paul E Brown PhD [94], Dr Lily Tong PhD [48], Ms Alice Broos BSc [48], Mr Daniel Mair BSc [48], Mrs Jenna Nichols BSc [48], Dr Stephen N Carmichael PhD [48], Dr Katherine L Smollett PhD [40], Dr Kyriaki Nomikou PhD [48], Dr Elihu Aranday-Cortes PhD/DVM [48], Ms Natasha Johnson BSc [48], Dr Seema Nickbakhsh PhD [48, 68], Dr Edith E Vamos PhD [92], Dr Margaret Hughes PhD [92], Dr Lucille Rainbow PhD [92], Mr Richard Eccles MSc [92], Ms Charlotte Nelson MSc [92], Dr Mark Whitehead PhD [92], Dr Richard Gregory PhD [92], Mr Matthew Gemmell MSc [92], Ms Claudia Wierzbicki BSc [92], Ms Hermione J Webster BSc [92], Ms Chloe L Fisher MSc [28], Mr Adrian W Signell BSc [20], Dr Gilberto Betancor PhD [20], Mr Harry D Wilson BSc [20], Dr Gaia Nebbia PhD FRCPath [12], Dr Flavia Flaviani PhD [31], Mr Alberto C Cerda MSc [96], Ms Tammy V Merrill MSc [96], Rebekah E Wilson MSc [96], Mr Marius Cotic MSc [82], Miss Nadua Bayzid BSc [82], Dr Thomas Thompson PhD [72], Dr Erwan Acheson PhD [72], Prof Steven Rushton PhD [51], Prof Sarah O'Brien PhD [51], David J Baker BEng [70], Steven Rudder [70], Alp Aydin MSci [70], Dr Fei Sang PhD [18], Dr Johnny Debebe PhD [18], Dr Sarah Francois PhD [23], Dr Tetyana I Vasylyeva DPhil [23], Dr Marina Escalera Zamudio PhD [23], Mr Bernardo Gutierrez MSc [23], Dr Angela Marchbank BSc [10], Joshua Maksimovic FD [9], Karla Spellman FD [9], Kathryn McCluggage Msc [9], Dr Mari Morgan PhD [69], Robert Beer BSc [9], Safiah Afifi BSc [9], Trudy Workman HNC [10], William Fuller BSc [10], Catherine Bresner Bsc [10], Dr Adrienn Angyal PhD [93], Dr Luke R Green PhD [93], Dr Paul J Parsons PhD [93], Miss Rachel M Tucker MSc [93], Dr Rebecca Brown PhD [93] and Mr Max Whiteley PhD [93]

Software and analysis tools:
James Bonfield BSc [99], Dr Christoph Puethe [99], Mr Andrew Whitwham BSc [99], Jennifier Liddle [99], Dr Will Rowe PhD [41], Dr Igor Siveroni PhD [39], Dr Thanh Le-Viet PhD [70] and Amy Gaskin MSc [69].

Visualisation:
Dr Rob Johnson PhD [39].

1 Barking, Havering and Redbridge University Hospitals NHS Trust, **2** Basingstoke Hospital, **3** Belfast Health & Social Care Trust, **4** Betsi Cadwaladr University Health Board, **5** Big Data Institute, Nuffield Department of Medicine, University of Oxford, **6** Brighton and Sussex University Hospitals NHS Trust, **7** Cambridge Stem Cell Institute, University of Cambridge, **8** Cambridge University Hospitals NHS Foundation Trust, **9** Cardiff

and Vale University Health Board, **10** Cardiff University, **11** Centre for Clinical Infection & Diagnostics Research, St. Thomas' Hospital and Kings College London, **12** Centre for Clinical Infection and Diagnostics Research, Department of Infectious Diseases, Guy's and St Thomas' NHS Foundation Trust, **13** Centre for Enzyme Innovation, University of Portsmouth (PORT), **14** Centre for Genomic Pathogen Surveillance, University of Oxford, **15** Clinical Microbiology Department, Queens Medical Centre, **16** Clinical Microbiology, University Hospitals of Leicester NHS Trust, **17** County Durham and Darlington NHS Foundation Trust, **18** Deep Seq, School of Life Sciences, Queens Medical Centre, University of Nottingham, **19** Department of Infection Biology, Faculty of Infectious & Tropical Diseases, London School of Hygiene & Tropical Medicine, **20** Department of Infectious Diseases, King's College London, **21** Department of Microbiology, Kettering General Hospital, **22** Departments of Infectious Diseases and Microbiology, Cambridge University Hospitals NHS Foundation Trust; Cambridge, UK, **23** Department of Zoology, University of Oxford, **24** Division of Virology, Department of Pathology, University of Cambridge, **25** East Kent Hospitals University NHS Foundation Trust, **26** East Suffolk and North Essex NHS Foundation Trust, **27** Gateshead Health NHS Foundation Trust, **28** Genomics Innovation Unit, Guy's and St. Thomas' NHS Foundation Trust, **29** Gloucestershire Hospitals NHS Foundation Trust, **30** Great Ormond Street Hospital for Children NHS Foundation Trust, **31** Guy's and St. Thomas' BRC, **32** Guy's and St. Thomas' Hospitals, **33** Hampshire Hospitals NHS Foundation Trust, **34** Health Data Research UK Cambridge, **35** Health Services Laboratories, **36** Heartlands Hospital, Birmingham, **37** Hub for Biotechnology in the Built Environment, Northumbria University, **38** Imperial College Hospitals NHS Trust, **39** Imperial College London, **40** Institute of Biodiversity, Animal Health & Comparative Medicine, **41** Institute of Microbiology and Infection, University of Birmingham, **42** King's College London, **43** Liverpool Clinical Laboratories, **44** Maidstone and Tunbridge Wells NHS Trust, **45** Manchester University NHS Foundation Trust, **46** Microbiology Department, Wye Valley NHS Trust, Hereford, **47** MRC Biostatistics Unit, University of Cambridge, **48** MRC-University of Glasgow Centre for Virus Research, **49** National Infection Service, PHE and Leeds Teaching Hospitals Trust, **50** Newcastle Hospitals NHS Foundation Trust, **51** Newcastle University, **52** NHS Greater Glasgow and Clyde, **53** NHS Lothian, **54** Norfolk and Norwich University Hospital, **55** Norfolk County Council, **56** North Cumbria Integrated Care NHS Foundation Trust, **57** North Tees and Hartlepool NHS Foundation Trust, **58** Northumbria University, **59** Oxford University Hospitals NHS Foundation Trust, **60** PathLinks, Northern Lincolnshire & Goole NHS Foundation Trust, **61** Portsmouth Hospitals University NHS Trust, **62** Princess Alexandra Hospital Microbiology Dept., **63** Public Health Agency, **64** Public Health England, **65** Public Health England, Clinical Microbiology and Public Health Laboratory, Cambridge, UK, **66** Public Health England, Colindale, **67** Public Health England, Colindale, **68** Public Health Scotland, **69** Public Health Wales NHS Trust, **70** Quadram Institute Bioscience, **71** Queen Elizabeth Hospital, **72** Queen's University Belfast, **73** Royal Devon and Exeter NHS Foundation Trust, **74** Royal Free NHS Trust, **75** Sandwell and West Birmingham NHS Trust, **76** School of Biological Sciences, University of Portsmouth (PORT), **77** School of Pharmacy and Biomedical Sciences, University of Portsmouth (PORT), **78** Sheffield Teaching Hospitals, **79** South Tees Hospitals NHS Foundation Trust, **80** Swansea University, **81** University Hospitals Southampton NHS Foundation Trust, **82** University College London, **83** University Hospital Southampton NHS Foundation Trust, **84** University Hospitals Coventry and Warwickshire, **85** University of Birmingham, **86** University of Birmingham Turnkey Laboratory, **87** University of Brighton, **88** University of Cambridge, **89** University of East Anglia, **90** University of Edinburgh, **91** University of Exeter, **92** University of Liverpool, **93** University of Sheffield, **94** University of Warwick, **95** University of Cambridge, **96** Viapath, Guy's and St Thomas' NHS Foundation Trust, and King's College Hospital NHS Foundation Trust, **97** Virology, School of Life Sciences, Queens Medical Centre, University of Nottingham, **98** Wellcome Centre for Human Genetics, Nuffield Department of Medicine, University of Oxford, **99** Wellcome Sanger Institute, **100** West of Scotland Specialist Virology Centre, NHS Greater Glasgow and Clyde, **101** Department of Medicine, University of Cambridge, **102** Ministry of Health, Sri Lanka, **103** NIHR Health Protection Research Unit in HCAI and AMR, Imperial College London, **104** North West London Pathology, **105** NU-OMICS, Northumbria University, **106** University of Kent, **107** University of Oxford, **108** University of Southampton, **109** University of Southampton School of Health Sciences, **110** University of Southampton School of Medicine, **111** University of Surrey, **112** Warwick Medical School and Institute of Precision Diagnostics, Pathology, UHCW NHS Trust, **113** Wellcome Africa Health Research Institute Durban and **114** Wellcome Genome Campus.

AMPHEUS Project

Oxford Viral Sequencing Group, Wellcome Centre for Human Genetics, University of Oxford, UK

Christophe Fraser

David Buck

Angie Green

George MacIntyre-Cockett

Paolo Piazza

John A Todd

Amy Trebes

Oxford Viral Sequencing Group, Big Data Institute, University of Oxford, UK

Laura Thomson

Acknowledgements

**Advent, South Africa**
Michael Breese
**Aneurin Bevan University Health Board**
Catherine Bailey
Jessica Harris
**BioIndustry Association**
Annette England
Ian McCubbin
**Cell & Gene Therapy Catapult**
Stephen Ward
**CobraBio**
**Clinical Trials Research Governance Office, University of Oxford**
Ronja Bahadori
Elaine Chick
Heather House
Claire Riddle
**Data and Safety Monitoring Board (DSMB)**
George Bouliotis
Steve Black
Elizabeth Bukusi
Cornelia Dekker
Robert Heyderman

Gregory Hussey
Paul Kaye
Bernhards Ogutu
Walter Orenstein
Sonia Ramos
Manish Sadarangani
**Deloitte UK**
Alex Hope
**Department of Health and Social Care, UK Government**
Amina Elmi
Harry Mayhew
Martin Shanahan
**Department of Paediatrics, University of Oxford**
Joanna Bagniewska
Elizabeth Derow
Georg A. Holländer
Samantha Vanderslott
**Endpoint Evaluation Committee**
Jeremy Carr
Stephen Chambers
Kim Davis
Simon Drysdale
Malick Gibani
Elizabeth Hammershaimb
Michael Harrington
Celina Jin
Seilesh Kadambari
Rama Kandasamy
Toby Maher
Jamilah Meghji
Claire Munro[1]
David Pace
Rekha R. Rapaka
Robindra Basu Roy
Daniel Silman
Gemma Sinclair
Jing Wang

**Halix**
**The Cambridge NIHR CRF COVID Vaccine Group**
**The GSTT NIHR CRF COVID Vaccine Group**
**The Imperial CRF COVID Vaccine Group**
**Jenner Institute, University of Oxford**
Iona Tarbet
**Marie Bashir Institute for Infectious Diseases and Biosecurity, University of Sydney**
Rebecca J Rockett
Vitali Sintchenko
**Nuffield Department of Medicine, University of Oxford**
Richard Cornall
Richard Liwicki
Denis Murphy
Elizabeth Salter
Katherine Skinner
Philip Taylor
Oto Velicka
**Oxford Biomedica**
**Oxford Research Services (Contracts)**
Carly Banner
Sally Pelling-Deeves
Gary Priest
**Oxford University Hospitals NHS Trust**
Monique Andersson
Laura Dunn
Bruno Holthof
**Pall Europe**
**Public Affairs Directorate and Divisional Communication Team**
Alison Brindle
Alexander Buxton
James Colman
Chris McIntyre
Steve Pritchard
**Sartorius**
Zander Hack
**VMIC**
